# FABP5 regulates an immunosuppressive microenvironment in hepatocellular carcinoma through the NF-κB/PD-L1 axis

**DOI:** 10.1371/journal.pone.0356359

**Published:** 2026-09-29

**Authors:** Rui An, Shibing Hou, Min Hong, Jiaorong Zhen, Zhijuan Wang, Haoming Huang, Weixuan Zhao, Mianmian Liao, Weiqu Yuan, Danping Huang

**Affiliations:** 1 The First Affiliated Hospital of Guangdong Pharmaceutical University, Guangzhou, Guangdong, People’s Republic of China; 2 The Affiliated Dongguan Songshan Lake Central Hospital, Guangdong Medical University, Dongguan, Guangdong, China; 3 Department of Acupuncture, Shenzhen Traditional Chinese Medicine Hospital, The Fourth Clinical Medical College of Guangzhou University of Chinese Medicine, Shenzhen, Guangdong, China; 4 Department of Radiology, The First Affiliated Hospital of Guangzhou University of Chinese Medicine, Guangzhou, Guangdong, China; 5 Dongguan Key Laboratory of Traditional Chinese Medicine Hepatology, the Affiliated Dongguan Songshan Lake Central Hospital, Guangdong Medical University, Dongguan, China; Charite Universitatsmedizin Berlin, GERMANY

## Abstract

**Background:**

Fatty acid binding protein 5 (FABP5) contributes to lipid metabolism and inflammation, but, its immunoregulatory role in hepatocellular carcinoma (HCC) is unclear. Considering the strong metabolic–immune interplay in HCC, elucidating how FABP5 shapes the tumor immune microenvironment may uncover new therapeutic targets.

**Methods:**

TCGA, CCLE and single-cell RNA-seq datasets were employed to characterize FABP5 expression and clinical relevance. FABP5 knockdown was performed to assess effects on HCC cell proliferation. Immune infiltration, immune checkpoint profiles, TIDE scores and enrichment of immune-related pathways were analyzed. Differentially expressed genes underwent GO and KEGG pathway enrichment analyses. Protein–protein docking, molecular dynamics (MD) simulations and in vitro assays were conducted to explore the regulatory role of FABP5 in the NF-κB/PD-L1 axis. Drug screening, molecular docking and duplicate MD simulations were carried out to identify potential FABP5-targeting compounds.

**Results:**

FABP5 was significantly upregulated in HCC and predicted worse survival, serving as an independent prognostic factor. Single-cell analysis identified predominant FABP5 expression in fibroblasts and macrophages. FABP5 knockdown suppressed cell proliferation, colony formation, DNA synthesis and migration, accompanied by decreased expression of epithelial–mesenchymal transition (EMT) markers. FABP5-high tumors exhibited increased immune infiltration but raised immune checkpoint levels and higher TIDE scores, indicative of an immunosuppressive microenvironment. FABP5 correlated positively with NF-κB pathway genes and molecular docking predicted stable complexes between IκBα and P65. Experimental validation confirmed that FABP5 drives PD-L1 upregulation in an NF-κB–dependent manner. Drug screening identified CZS-241 as the most promising FABP5-binding candidate, and duplicate MD simulations demonstrated a stable FABP5–CZS-241 complex.

**Conclusion:**

FABP5 drives immunosuppression in HCC by inducing PD-L1 via the NF-κB pathway. CZS-241 is identified as a promising FABP5-targeting compound with therapeutic potential for HCC.

## 1. Introduction

Liver cancer remains one of the most prevalent and lethal malignancies worldwide, with significant geographical and demographic disparities. First, according to GLOBOCAN data from 2022, there were approximately 866,136 new cases of liver cancer and 758,725 related deaths globally, with a higher burden observed in males and the elderly [1]. Second, by 2040, the annual number of new cases is projected to rise to approximately 1.39 million [2]. This trend suggests that despite the ongoing decline in the seroprevalence of hepatitis B virus (HBV) in some high-risk countries, the growing prevalence of obesity and diabetes may reverse the progress in controlling liver cancer [3]. Third, the etiology of liver cancer is also evolving. While hepatitis B remains the leading cause of liver cancer globally, metabolic dysfunction-associated steatotic liver disease (MASLD) is the sole factor that has seen both increasing incidence and mortality rates [4].

Fatty acid-binding protein 5 (FABP5) is an intracellular carrier involved in lipid transport and metabolism [5]. While its role in MASLD has been noted, its function in hepatocellular carcinoma (HCC), particularly within the tumor immune microenvironment (TIME), remains incompletely understood [6,7].

Emerging evidence has begun to position FABP5 as a key node linking metabolic reprogramming to immune regulation. In HCC, tumor-derived exosomal FABP5 can remodel the TIME by inducing lipid accumulation and M2 polarization in macrophages [8]. In malignant pleural effusion, C1q+ tumor-associated macrophages utilize FABP5-driven fatty acid metabolism to promote immunosuppression [9]. Beyond the myeloid compartment, FABP5 also supports CD8 + T cell antitumor function by facilitating fatty acid uptake, a process impaired in the tumor microenvironment [10]. Furthermore, FABP5 has been shown to activate pro-inflammatory NF-κB signaling and to induce PPARγ-mediated lipid metabolic reprogramming in other cancers [11,12]. In summary, FABP5 plays a crucial role in the pathogenesis of HCC and could serve as a novel therapeutic target. Future research should further explore the specific mechanisms of FABP5 in these diseases to develop more effective treatment strategies [13].

In this study, we comprehensively evaluated the expression patterns and prognostic significance of FABP5 across multiple malignancies using TCGA, CCLE, and other public databases, with a specific focus on LIHC. We assessed the correlation between FABP5 expression and key clinical characteristics in LIHC patients. To investigate the potential role of FABP5 in the tumor immune microenvironment, we analyzed single-cell RNA sequencing (scRNA-seq) data to map its expression across diverse cellular compartments within LIHC tissues. We further analyzed the association between FABP5 expression levels and tumor-infiltrating immune cell abundance using the CIBERSORT algorithm [14]. The relationship between FABP5 expression and immune checkpoint molecule expression was also systematically investigated. Additionally, we utilized TIDE scoring to evaluate the potential link between FABP5 expression and tumor immune evasion. Gene Ontology (GO) and Kyoto Encyclopedia of Genes and Genomes (KEGG) pathway enrichment analyses were performed on differentially expressed genes (DEGs) between FABP5-high and FABP5-low expression groups to elucidate potential biological pathways and immune-related functions associated with FABP5 activity. A prognostic nomogram incorporating FABP5 expression and clinical stage was constructed to predict 1-, 3-, and 5-year survival probabilities for LIHC patients. Furthermore, we conducted *in vitro* experiments involving FABP5 knockdown in HCC cell lines to assess its functional impact on cellular proliferation and inflammation-associated pathways. Finally, molecular docking and duplicate molecular dynamics (MD) simulations were used to explore the binding interactions and stability features between FABP5 and potential drugs. Collectively, our findings position FABP5 not only as a prognostic biomarker and oncogenic driver in LIHC, but also as a key regulator of the immunosuppressive tumor microenvironment, underscoring its dual potential as a therapeutic target for both tumor cells and immune evasion mechanisms.

## 2. Materials and methods

### 2.1. Data acquisition and preprocessing

We acquired the STAR-counts data of LIHC tumors and the corresponding clinical information from The Cancer Genome Atlas (TCGA, https://portal.gdc.cancer.gov). Transcript-level expression values were converted into TPM format, followed by log2(TPM + 1) normalization. After retaining only samples with both RNA-seq data and clinical information, a total of 10,228 samples were included for pan-cancer analysis, among which 371 LIHC samples were used for subsequent liver cancer–specific analyses. Gene expression profiles of liver cancer cell lines were retrieved from the Cancer Cell Line Encyclopedia (CCLE, https://depmap.org/portal/data_page/?tab=allData). All bioinformatic analyses in this study were conducted using the ACLBI platform (https://www.aclbi.com/), which provides a unified graphical interface for running standard R-based computational pipelines. Although the analyses were executed through the online platform, ACLBI implements well-established algorithms built on publicly available R packages, ensuring methodological transparency and reproducibility. All statistical analyses were carried out using R software (version 4.0.3). A *p*-value < 0.05 was considered statistically significant. Protein expression levels of FABP5 in LIHC were retrieved from the Human Protein Atlas (HPA, http://www.proteinatlas.org), which provides immunohistochemistry (IHC)-based expression profiles across multiple cancer types.

### 2.2. Prognostic analysis in Pan-Cancer Cohort

Prognostic analyses across cancers were performed using Kaplan-Meier survival curves. The corresponding *p*-values and hazard ratios (HRs) with 95% confidence intervals (CIs) were calculated using log-rank tests and univariate Cox regression analyses.

### 2.3. Single-Cell RNA Sequencing Analysis

To investigate the expression patterns of FABP5 across distinct cell populations in HCC, published single-cell RNA sequencing (scRNA-seq) datasets were analyzed. The scRNA-seq raw data (.h5 format) and corresponding annotation files were downloaded from the Tumor Immune Single-cell Hub (TISCH) database. Data processing and downstream analyses were conducted using the MAESTRO and Seurat R packages. Dimensionality reduction and cell clustering were performed with the t-distributed stochastic neighbor embedding (t-SNE) algorithm, allowing visualization of FABP5 expression levels across different cellular subsets.

### 2.4. Prognostic nomogram construction

Candidate prognostic variables were initially identified by univariate and multivariate Cox regression analyses. Variables with significant prognostic value in both analyses were considered independent prognostic factors. A prognostic nomogram was subsequently constructed using the “rms” R package to predict 1-, 3-, and 5-year overall survival (OS) probabilities. Calibration curves were plotted to evaluate the predictive accuracy of the model. Forest plots were generated using the “forestplot” R package to visually display the *p*-values, HRs, and 95% CIs of each variable.

### 2.5. Cell culture and transfection

The HCC cell line Huh7 was obtained from the American Type Culture Collection (ATCC, Manassas, VA, USA). Cells were cultured in Dulbecco’s Modified Eagle’s Medium (DMEM; Gibco) supplemented with 10% fetal bovine serum (FBS; Gibco) under standard conditions (37 °C, 5% CO_2_). FABP5 knockdown: FABP5-siRNA plasmids (siFABP5#1 and siFABP5#2) and a negative control siRNA plasmid (siNC) were purchased from GeneChem (Shanghai, China). The targeting sequences were as follows: siFABP5#1, sense strand: 5’-AGGAGUUAAUUAAGAGAAUGATT-3’; antisense strand: 5’-UCAUUCUCUUAAUUAACUCCUTT-3’; siFABP5#2: sense strand: 5’-UGAAUACAUGAAGGAGCUATT-3’; antisense strand: 5’-UAGCUCCUUCAUGUAUUCATT-3.’ For transfection, Huh7 cells were seeded in 6-well plates at approximately 80% confluence and transfected with 2 µg of plasmid using Lipofectamine 3000 (Invitrogen, Carlsbad, CA, USA). After 6–8 h of incubation, the transfection medium was replaced with complete culture medium, and cells were collected after 48 h for subsequent experiments.

FABP5 overexpression: The full-length human FABP5 coding sequence was amplified and inserted into the pCDNA3.1(+) expression vector (Invitrogen, Carlsbad, CA, USA). The empty pCDNA3.1(+) vector was used as a negative control. For transfection, Huh7 cells were seeded in 6-well plates and cultured overnight until they attained approximately 70–80% confluence. Cells were then transfected with 2 µg of either FABP5-overexpressing plasmid (pcDNA-FABP5) or empty vector (pcDNA-NC) using Lipofectamine 3000 (Invitrogen) according to the manufacturer’s protocol. After 6–8 h of incubation, the transfection mixture was replaced with fresh complete culture medium. Cells were harvested after 48 h for subsequent experiments. FABP5 overexpression was confirmed by Western blot.

### 2.6. Cell viability assay

Cell viability was assessed using the Cell Counting Kit-8 (CCK-8) assay (GLPBIO, GK1000). Huh7 cells in the logarithmic growth phase were seeded into 96-well plates at a density of 3,000 cells per well. CCK-8 reagent was supplemented daily for three consecutive days, and the absorbance was measured at 450 nm after 3 h of incubation at 37°C. Growth curves were plotted from absorbance values.

### 2.7. Colony formation assay

Cells were plated into 6-well plates at a density of 500 cells per well and cultured for three weeks with regular medium replacement every 2–3 days. Colonies were fixed with 4% paraformaldehyde (Biosharp, BL539A), stained with 0.1% crystal violet (Beyotime Biotechnology, C0121), and rinsed with phosphate-buffered saline (PBS). Images were captured, and colonies were quantified for statistical analysis.

### 2.8. EdU (5-Ethynyl-2-Deoxyuridine) proliferation assay

Cell proliferation was assessed using the EdU assay kit (APExBIO, K1076). Huh7 cells were seeded into 96-well plates at a density of 3 × 10^5^ cells/mL (100 µL per well) and incubated overnight to permit cell attachment. Upon reaching approximately 70% confluence, cells were incubated with 20 µM EdU for 3 h at 37°C. After EdU labeling, cells were fixed with 4% paraformaldehyde, permeabilized with 0.3% Triton X-100, and stained with Click Reaction Buffer and Hoechst 33342. Images were acquired with a fluorescence microscope, and the percentage of EdU-positive cells was quantified.

### 2.9. Western blot

Cellular proteins were extracted using RIPA buffer (NCM, WB3100) supplemented with protease inhibitors. Protein concentrations were determined using a BCA protein assay kit (CWBIO, CW0014S). Equal amounts of protein were denatured, separated by 8–12% SDS-PAGE, and transferred onto polyvinylidene difluoride (PVDF) or nitrocellulose membranes. The membranes were blocked with a blocking solution (Servicebio, CR2405137) for 1 hour at room temperature and then incubated with primary antibodies overnight at 4°C. After washing, the membranes were incubated with horseradish peroxidase (HRP)-conjugated secondary antibodies for 1 h at room temperature. Protein bands were detected using High-sig ECL Western blotting substrate (Tanon, 180–5001) and detected with a chemiluminescence imaging system (Servicebio).

The following antibodies were employed in this research: anti-β-actin (66009–1-IG, 1:3000; Proteintech), anti-E-cadherin (20874–1-AP, 1:1000; Proteintech), anti-N-cadherin (22018–1-AP, 1:1000; Proteintech), anti-Vimentin (10366–1-AP, 1:1000; Proteintech), anti-FABP5 (12348–1-AP, 1:3000; Proteintech),anti-IκBα (10268–1-AP, 1:10000; Proteintech),anti-p-IκBα (82349–1-RR, 1:2000; Proteintech),anti-PD-L1 (28076–1-AP, 1:1000; Proteintech),HRP-conjugated Goat Anti-Mouse IgG (H + L) (SA00001–1, 1:5000; Proteintech), HRP-conjugated Goat Anti-Rabbit IgG (H + L) (SA00001–2, 1:5000; Proteintech). All antibodies were diluted with Universal Antibody Diluent (NCM, WB500D) for dilution.

### 2.10. CIBERSORT analysis

The CIBERSORT algorithm (https://cibersort.stanford.edu/) was applied to estimate relative abundance of 22 immune cell subsets in each sample. FABP5 expression levels in each sample were extracted and Spearman correlation coefficients were calculated with relative abundance of the 22 immune cell types. Visualization of correlation heatmaps was performed using R package ggplot2 v3.4.0. The ESTIMATE algorithm (Wilkerson and Hayes, 2010) was utilized to calculate stromal, immune, and overall ESTIMATE scores between FABP5 high- and low-expression groups.

### 2.11. Analysis of immune checkpoint molecule expression

Immune checkpoint molecules serve as critical inhibitory regulators of the immune system that maintain self-tolerance and curb excessive immune activation. To compare immune checkpoint expression between high and low FABP5 expression groups, we evaluated a set of key immune checkpoint genes using the “limma” R package. These genes included ITPRIPL1, SIGLEC15, TIGIT, CD274 (PD-L1), HAVCR2 (TIM-3), PDCD1 (PD-1), CTLA4, LAG3, and PDCD1LG2 (PD-L2).

### 2.12. Functional enrichment analysis

Differentially expressed genes (DEGs) between FABP5 high- and low-expression groups were identified with the DESeq2 R package. Gene Ontology (GO) annotation and Kyoto Encyclopedia of Genes and Genomes (KEGG) pathway enrichment analyses were conducted using the clusterProfiler and org.Hs.e.g.,db R packages. Enrichment significance was set at *p* < 0.05.

### 2.13. Protein–protein docking of FABP5 and key proteins of the NF-κB signaling pathway

The three-dimensional crystal structures of FABP5 and key proteins of the NF-κB Signaling pathway (IκBα, IKKβ, p65) were retrieved from the Protein Data Bank (PDB). Prior to docking, the structures were preprocessed using PyMOL to remove water molecules, ligands, and heteroatoms, and to add missing hydrogen atoms. Protein–protein docking simulations were performed using the HDOCK web server (http://hdock.phys.hust.edu.cn/). The top-ranked docking conformations were selected based on the docking scores and cluster distributions. For each predicted complex, the binding interface and interacting residues were analyzed. Amino acid residues falling within 5 Å between the two proteins were identified as potential interaction sites. The binding conformations and interfacial residues were visualized and annotated using PyMOL.

### 2.14. Drug screening and molecular docking

The three-dimensional (3D) structure of FABP5 was obtained from the Protein Data Bank (PDB). Based on structural quality and suitability for molecular docking, 4azr was selected as the representative structure of FABP5 for subsequent molecular docking studies. We screened potential drugs that could target the FABP5 protein through the DGIdb (DrugGene Interaction Database) database and obtained the chemical structures of these drugs from the PubChem website (https://pubchem.ncbi.nlm.nih.gov/). Subsequently, molecular docking analysis was performed on the selected drugs and FABP5 protein using CB-Dock2 (https://cadd.labshare.cn/cb-dock2/php/index.php). The binding conformations were ranked according to their predicted binding free energy (kcal/mol). The best-docked poses were selected based on the lowest binding energy and favorable orientation within the active pocket.

### 2.15. Molecular dynamics simulation

In this study, molecular dynamics simulations were conducted using GROMACS 2022. Force field parameters for the receptor protein were generated using the pdb2gmx tool of GROMACS. For the ligand, topology files based on the GAFF2 force field were generated using the sobtop_1.0 (dev3.1) software, and atomic charges were assigned via the RESP method to ensure a charge distribution consistent with physicochemical properties. The AMBER14SB force field was employed for the receptor protein. During the simulation setup, the system was solvated using the TIP3P water model within a cubic water box of 1 nm to ensure adequate solvation and electrical neutrality. To maintain charge neutrality, Na⁺and Cl^−^ions were added to the system using the gmx genion tool in GROMACS, with an ion concentration of 0.15 M NaCl, mimicking conventional physiological ionic strength and charge neutralization conditions. Long‑range electrostatic interactions were handled using the Particle Mesh Ewald (PME) method with a cutoff distance of 1 nm. Force field parameters and PME settings were optimized according to GROMACS guidelines. Bond constraints were applied using the LINCS algorithm. Prior to the production molecular dynamics run, the system underwent an energy minimization procedure. Energy minimization consisted of 3000 steps of steepest descent followed by 2000 steps of conjugate gradient optimization. This process was divided into three stages: first, energy minimization with the solute constrained and water molecules relaxed; second, energy minimization with counterions constrained; and finally, full system energy minimization without constraints. During the simulation, the temperature was maintained at 310 K using the Nosé–Hoover thermostat, and the pressure was kept at 1 bar using the Parrinello–Rahman barostat. The simulation was carried out in the NPT ensemble for 100 ns with an integration time step of 2 fs. Throughout the simulation, GROMACS tools including gmx rmsd, gmx rmsf, gmx hbond, gmx Rg, and gmx sasa were used to calculate the root‑mean‑square deviation (RMSD), root‑mean‑square fluctuation (RMSF), hydrogen bonds (H‑bonds), radius of gyration (Rg), and solvent‑accessible surface area (SASA) and free energy landscapes (FEL), respectively. FEL were generated using RMSD and Rg as reaction coordinates to identify energetically favorable conformational states. These analyses were performed to evaluate system stability, structural changes, and solvent effects.

### 2.16. RNA extraction and quantitative real-time PCR (qPCR)

Total RNA was extracted from cultured cells using the EZ-press RNA Purification Kit (B0004D, EZBioscience, USA). RNA concentration and purity were determined using a NanoDrop 2000 spectrophotometer (Thermo Fisher Scientific, Waltham, MA, USA). Reverse transcription was performed using 1 μg of total RNA with the 4 × EZscript Reverse Transcription Mix II (with gDNA Remover) (EZB-RT2GQ, EZBioscience, USA). The reverse transcription reaction was carried out under the following conditions: 42°C for 15 min, followed by 95°C for 30 s. The resulting cDNA (L420-A, Bioer Technology Co., Ltd., Hangzhou, China) was used for subsequent qPCR.

Quantitative real-time PCR was performed using 2 × SYBR Green qPCR Master Mix (ROX2) (A0001-R2, EZBioscience, USA) on a real-time PCR system (M1324R, Shanghai Hongshi Medical Technology Co., Ltd., Shanghai, China). The thermal cycling protocol was as follows: initial denaturation at 95°C for 5 min, followed by 40 cycles of 95°C for 10 s and 60°C for 30 s. A melting curve analysis was performed to verify the specificity of the amplification products.

The relative mRNA expression levels of target genes were calculated using the 2^(-ΔΔCt) method, with GAPDH as the internal control. All reactions were performed in triplicate, and three independent biological replicates were conducted.

### 2.17. Wound-healing assay

Cells were seeded in 6-well plates at a density of 5 × 10^5^ cells per well and cultured in complete medium until they reached approximately 90% confluence. A straight scratch was created across the center of each well using a sterile 1 mL pipette tip. Detached cells were gently removed by washing twice with phosphate-buffered saline (PBS). The remaining cells were then incubated in serum-free medium to minimize cell proliferation during the assay. Images of the scratched area were taken at 0 h, 24 h, 48 h, and 72 h under an inverted microscope (Olympus CKX53, Tokyo, Japan) at 4 × magnification. Wound closure was quantified by measuring the remaining scratch area using ImageJ software, and the percentage of wound closure was calculated as: (migrated distance / initial scratch width) × 100%.

### 2.18. Bay 11–7082 treatment

Bay 11–7082 (an NF-κB inhibitor, catalog #B5556-10MG, Sigma-Aldrich, St. Louis, MO, USA) was dissolved in dimethyl sulfoxide (DMSO, catalog #D8418, Sigma-Aldrich) to prepare a 10 mM stock solution. The stock solution was aliquoted and stored at −20°C. For each experiment, an aliquot was thawed and diluted to the desired concentration with serum-free medium immediately before use.

Huh7 cells were seeded in 6-well plates at a density of 2 × 10^5^ cells per well and cultured in complete medium overnight. When cells reached approximately 70–80% confluence, the medium was replaced with fresh medium containing Bay 11–7082 (5 µM) or an equal volume of DMSO (final concentration ≤0.1%) as a vehicle control. Cells were then incubated for 24 h under standard culture conditions (37°C, 5% CO_2_).

To evaluate the effect of Bay 11–7082 on FABP5-mediated NF-κB activation, FABP5-overexpressing cells and control cells were treated with 5 µM Bay 11–7082 or DMSO for 24 h. After treatment, cells were harvested for Western blot analysis to assess the expression levels of p-IκBα, IκBα, and PD-L1.

### 2.19. Statistical analysis

All experiments were performed in triplicate unless otherwise specified. Data are presented as mean ± standard deviation (SD). Statistical analyses were conducted using SPSS (version 25.0), and figures were generated with GraphPad Prism (version 9.0). Two-tailed Student’s t-test was used for comparisons between two groups, and one-way ANOVA was applied for comparisons among three or more groups. A *p*-value < 0.05 was considered statistically significant.

## 3. Results

### 3.1. FABP5 expression is upregulated in multiple cancers and predicts poor prognosis

To comprehensively evaluate the expression pattern of FABP5 across various cancer types, we first performed a pan-cancer analysis based on the TCGA database (Fig 1A). The results revealed that FABP5 was differentially expressed in multiple cancers, with significant upregulation in the majority of cancer types examined (e.g., Liver Hepatocellular Carcinoma, LIHC; Cholangiocarcinoma, CHOL; Esophageal Carcinoma, ESCA; Kidney Renal Clear Cell Carcinoma, KIRC; Stomach Adenocarcinoma, STAD; *p* < 0.05). Further analysis of its expression in liver cancer cell lines (Fig 1B) showed heterogeneity, with higher expression levels observed in cell lines such as Huh-7, JHH-7, JHH-5, and SNU-878, and lower levels in SNU-449, JHH-1, and SNU-761. We next analyzed the protein expression of FABP5 in liver cancer and normal liver tissues using the HPA database. The results showed that FABP5 expression was significantly higher in liver cancer tissues compared to normal liver tissues (Fig 1C). Subsequent Cox regression analysis of pan-cancer survival data from TCGA (Fig 1D) indicated that high FABP5 expression was significantly associated with worse overall survival (OS) in multiple cancer types, including Adrenocortical carcinoma (ACC), Lower Grade Glioma (LGG), LIHC, and Uveal Melanoma (UVM). Focusing on LIHC, we further analyzed the differential expression of FABP5 between tumor and normal tissues. The results demonstrated that FABP5 expression was significantly higher in liver cancer tissues compared to normal liver tissues (Fig 1E, *p* = 5.18 × 10 ^−20^). Paired sample analysis further confirmed this finding, showing that FABP5 expression was markedly higher in paired tumor tissues than in corresponding adjacent normal tissues (*p* < 0.001; Fig 1F). In summary, FABP5 is differentially expressed across multiple cancer types, exhibits significant upregulation in hepatocellular carcinoma, and is closely associated with patient survival outcomes.

**Fig 1:**
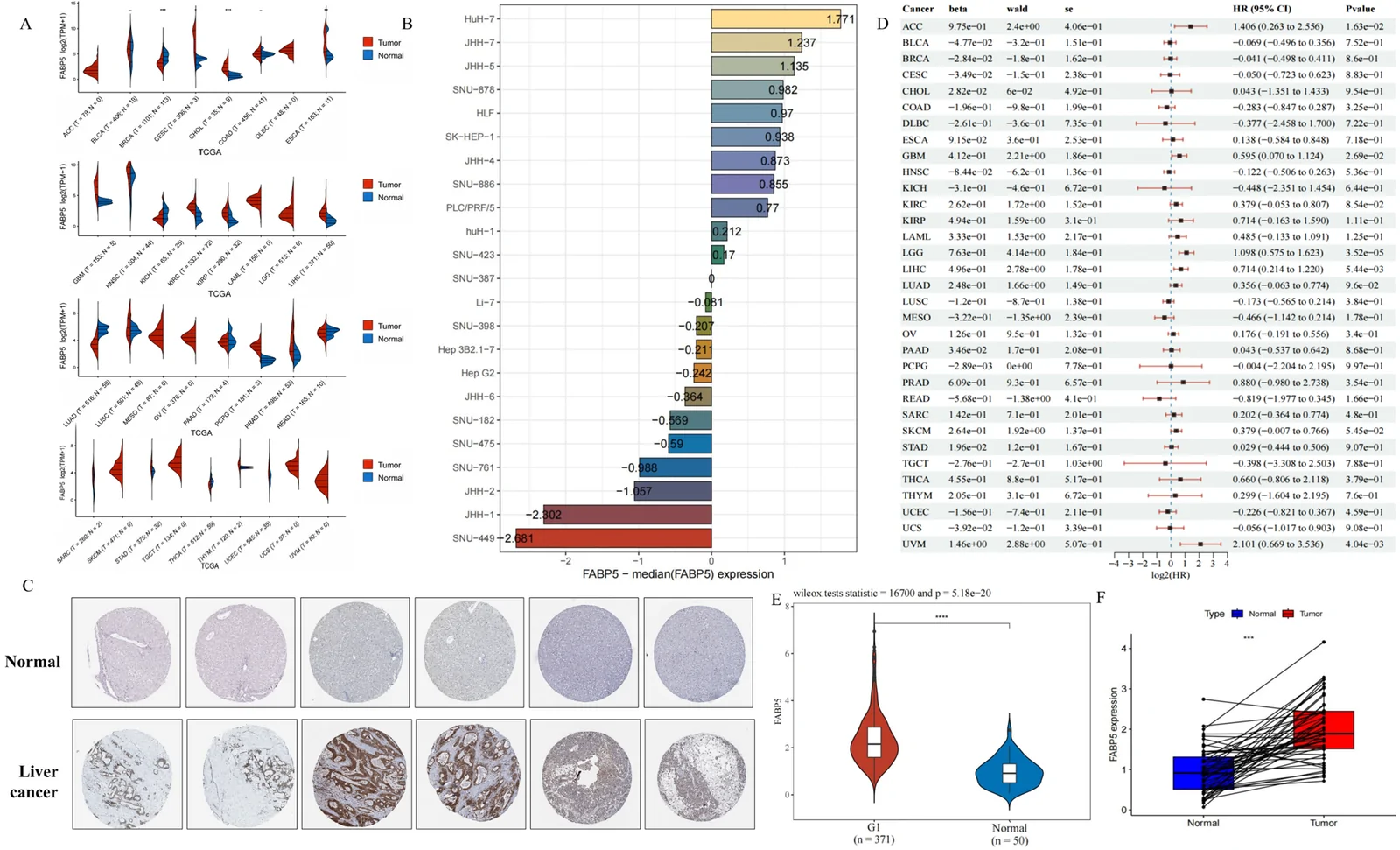
FABP5 is upregulated in multiple cancers and predicts poor prognosis. (A) Pan-cancer analysis of FABP5 expression based on TCGA datasets revealed significant differential expression across diverse tumor types. (B) FABP5 expression in HCC cell lines from the CCLE database showed heterogeneity across different lines. (C) Immunohistochemistry analysis from HPA database showing elevated FABP5 expression in HCC tissues compared with normal liver tissues. (D) Forest plot of Cox regression analyses showing FABP5 as a prognostic factor in multiple cancers. (E) FABP5 expression was significantly higher in HCC tissues compared with normal liver tissues. (F) Paired sample analysis further confirmed elevated FABP5 expression in HCC tumor tissues relative to adjacent non-tumor tissues. *p < 0.05, **p < 0.01, ***p < 0.001.

### 3.2. Single-cell transcriptomic analysis identifies stromal and immune cells as major sources of FABP5 expression

To characterize the cell type-specific expression of FABP5 in HCC tissues, we systematically analyzed single-cell RNA sequencing data from 19 HCC patients (approximately 9000 cells in total). As shown in Fig 2A, t-SNE dimensionality reduction and clustering analysis identified eight major cell subtypes: fibroblasts, monocytes/macrophages (Mono/Macro), endothelial cells, exhausted CD8 ⁺ T cells (CD8 ⁺ Tex), malignant cells, B cells, plasma cells, and hepatic progenitor cells. The distribution of FABP5 expression across these cell types is displayed in Fig 2B, where the color depth in the t-SNE plot reflects FABP5 expression abundance. Overall, FABP5 was detectable in multiple cell types, but its expression levels varied considerably. Further quantitative analysis of the average FABP5 expression levels across cell populations (Fig 2C) revealed that the cell types with the most prominent FABP5 expression were, in descending order: fibroblasts > monocytes/macrophages > endothelial cells > CD8 ⁺ Tex > malignant cells > B cells > plasma cells > hepatic progenitor cells. This finding suggests that FABP5 may primarily function within tumor stromal-related cells, particularly exhibiting high activity in fibroblasts and immune cells, indicating its potential role in regulating the tumor microenvironment, immune modulation, and stromal remodeling.

**Fig 2:**
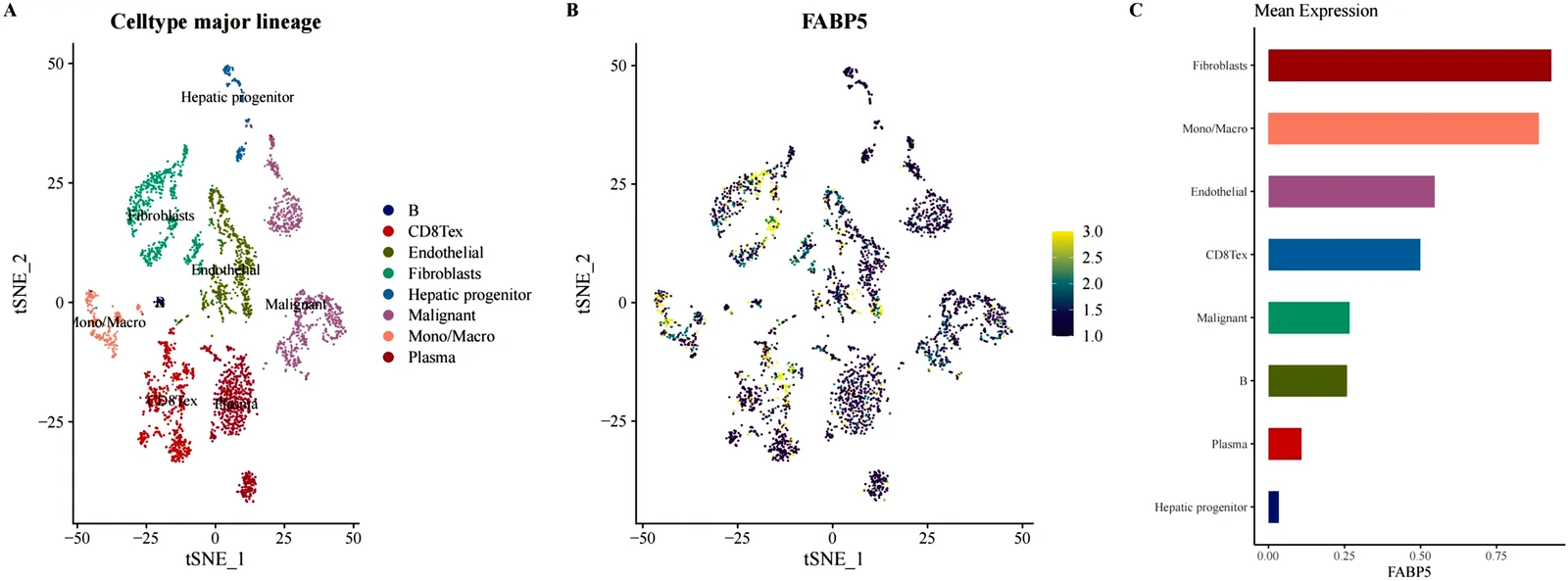
Single-cell transcriptomic profiling identifies stromal and immune cells as major sources of FABP5 expression in HCC. (A) t-SNE clustering of single-cell RNA sequencing data from 19 HCC patients identified eight major cellular subtypes. (B) Distribution of FABP5 expression across distinct cell clusters, with color intensity indicating relative abundance. (C) Average FABP5 expression across cell populations revealed highest levels in fibroblasts, macrophages, and endothelial cells.

### 3.3. High FABP5 expression correlates with distinct clinical features and TP53 mutation

Based on the TCGA database, we analyzed the distribution of clinical characteristics among HCC patients stratified by FABP5 expression levels (Figs 3A–3D). The distribution of FABP5 expression across different age groups showed significant differences (*p* = 0.005). The proportion of patients in the high FABP5 expression group (G1) was significantly higher in the > 61 years age group compared to the ≤ 61 years group (57% vs. 43%; Fig 3A). Significant differences in the distribution of high and low FABP5 expression were also observed among different racial groups (Fig 3B), with a notably higher proportion of high FABP5 expression among White individuals (57%). Regarding tumor grade (Fig 3C), the proportion of high FABP5 expression varied significantly across different grades: it was higher in patients with grade 1 tumors (65% vs. 35%, *p* = 0.022) and lower in those with grade 3 tumors (39% vs. 61%, *p* = 0.019), while no statistically significant differences were observed in grade 2 and grade 4 patients. A Sankey diagram (Fig 3D) comprehensively illustrates the relationships between FABP5 expression levels and clinical features, including patient survival status, tumor grade, race, and age. Furthermore, given that TP53 is a critical tumor suppressor gene whose mutation leads to loss of growth inhibitory function in tumor cells, we compared the TP53 mutation rate between different FABP5 expression groups (Fig 3E). The results revealed that the TP53 mutation rate was as high as 40% in the high FABP5 expression group, significantly higher than the 19% rate in the low expression group, with a statistically significant difference between the groups (*p* = 1.51 × 10 ^−5^).

**Fig 3:**
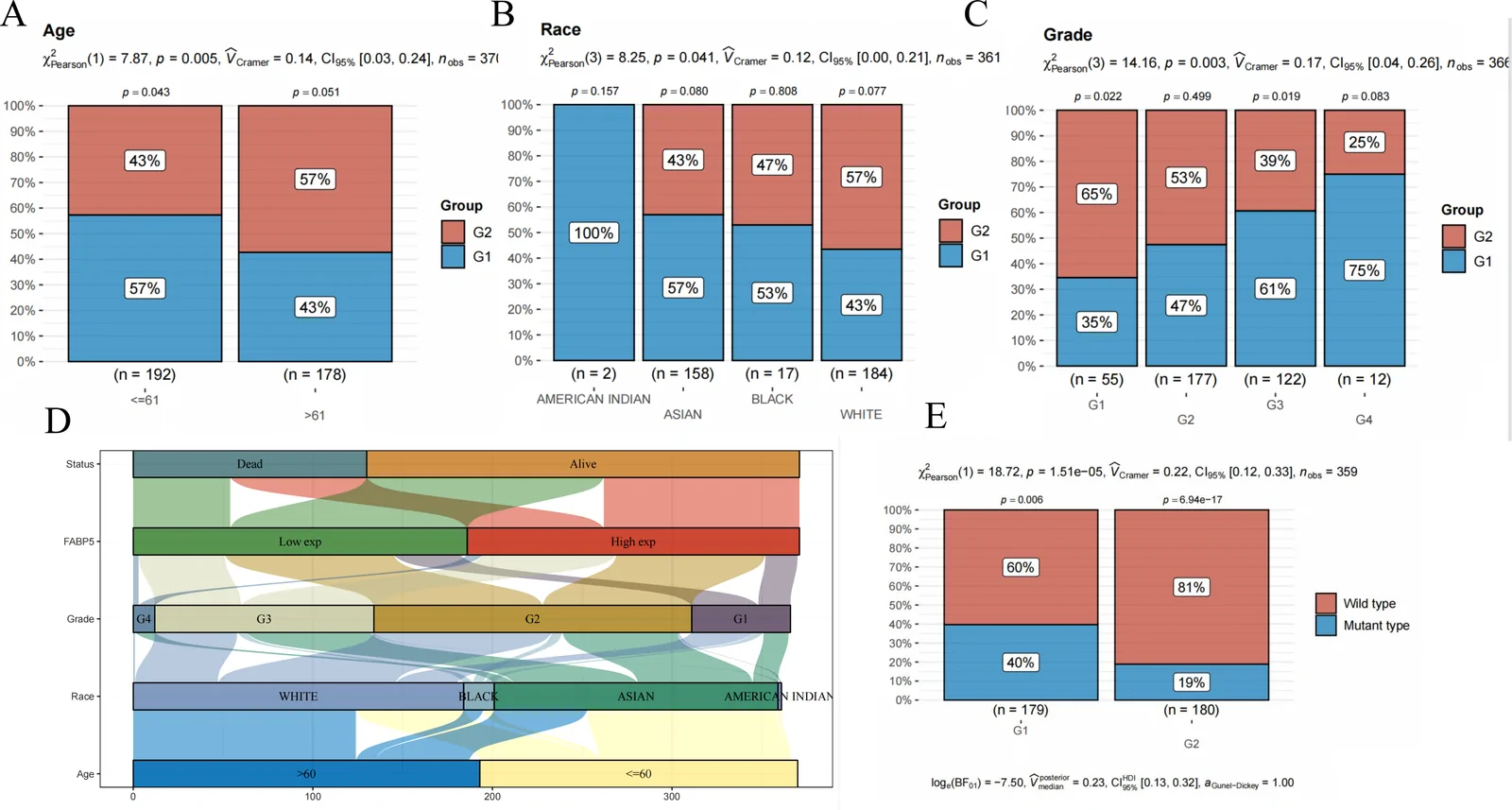
Clinical characteristics associated with FABP5 expression in HCC. (A) Distribution of FABP5 expression across different age groups. (B) FABP5 expression levels in patients stratified by race. (C) Association of FABP5 expression with tumor grade. (D) Sankey diagram summarizing relationships among FABP5 expression, patient survival status, tumor grade, race, and age. (E) TP53 mutation frequency was significantly higher in patients with high FABP5 expression compared with those with low expression. G1: Group 1 (High Expression); G2: Group 2 (Low Expression).

### 3.4. Elevated FABP5 expression serves as an independent prognostic factor for adverse outcomes in LIHC

Within the TCGA-LIHC cohort, we analyzed the relationship between FABP5 expression levels and patient prognosis (Figs 4A and 4B). Kaplan-Meier survival curves revealed that patients in the high FABP5 expression group had significantly worse overall survival (OS) than those in the low expression group (Fig 4A, Log-rank *p* = 0.00544, HR = 1.642, 95% CI: 1.157–2.328). Disease-specific survival (DSS) was also significantly reduced in the high expression group (Fig 4B, Log-rank *p* = 0.00202, HR = 2.058, 95% CI: 1.301–3.255), indicating that high FABP5 expression is associated with poor prognosis in liver cancer patients. Univariate Cox regression analysis (Fig 4C) showed that FABP5 expression and advanced pTNM stage (Stage III/IV vs. Stage I) were significantly associated with worse prognosis (*p* < 0.05), while tumor grade (G2, G3, G4 vs. G1) did not reach statistical significance in this cohort (*p* > 0.05). FABP5 expression was significantly associated with an increased risk of death in the univariate Cox model (HR = 1.348, 95% CI: 1.159–1.568, *p* = 1.09 × 10^-4^). Multivariate Cox regression analysis (Fig 4D) further confirmed that FABP5 expression remained independently associated with poor prognosis after adjustment for clinicopathological variables (HR = 1.420, 95% CI: 1.144–1.763, *p* = 1.47 × 10^-3^). Based on the multivariate analysis results, we constructed a prognostic nomogram integrating FABP5 expression level and pTNM stage to predict 1-, 3-, and 5-year survival probabilities (Fig 4E). The nomogram demonstrated a C-index of 0.715 (95% CI: 0.649–0.781, *p* < 0.001), indicating good predictive accuracy. The calibration curve (Fig 4F) showed a high consistency between the nomogram-predicted survival probabilities and actual observations, with particularly good agreement for the 3- and 5-year survival predictions.

**Fig 4:**
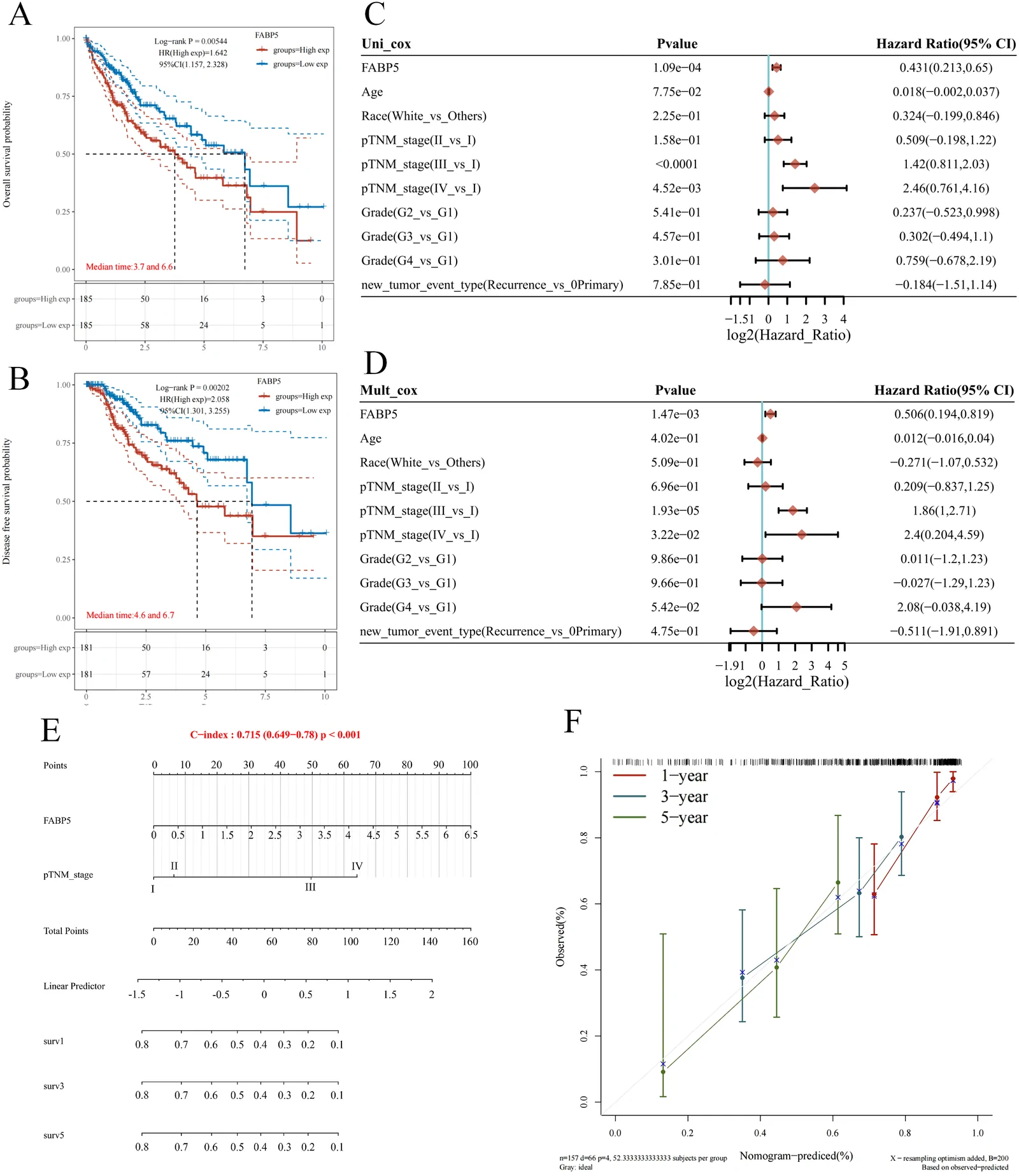
High FABP5 expression predicts poor prognosis and serves as an independent prognostic factor in HCC. (A) Kaplan–Meier survival curves showing that high FABP5 expression is associated with reduced overall survival. (B) Kaplan–Meier survival analysis for disease-specific survival revealed significantly worse outcomes in the high FABP5 group. (C) Univariate Cox regression analysis identified FABP5 expression and TNM stage as significant prognostic factors. (D) Multivariate Cox regression confirmed FABP5 as an independent prognostic factor. (E) Prognostic nomogram integrating FABP5 expression and TNM stage to predict 1-, 3-, and 5-year survival. (F) Calibration plots showing consistency between predicted and observed survival probabilities.

### 3.5. FABP5 knockdown suppresses HCC cell proliferation in vitro

Given that prior analysis indicated the highest expression of FABP5 in Huh7 cells, we selected this cell line for subsequent functional experiments. qPCR results (Fig 5A) showed that transfection with si-FABP5#1 and si-FABP5#2 significantly reduced FABP5 mRNA levels (*p* < 0.001), indicating effective knockdown. The CCK-8 assay (Fig 5B) revealed that FABP5 knockdown significantly decreased the viability of Huh7 cells at 24, 48, and 72 hours, indicating markedly inhibited cell growth compared to the control group. The colony formation assay (Fig 5C) further confirmed that the number of colonies formed in the si-FABP5#1 and si-FABP5#2 treatment groups was significantly lower than that in the control group (*p* < 0.001). EdU assay results (Fig 5D) demonstrated that FABP5 knockdown significantly reduced the proportion of EdU-positive Huh7 cells (*p* < 0.001), indicating a substantial decrease in cell proliferation capacity. The scratch wound assay revealed that the wound healing capability of Huh7 cells was markedly suppressed after FABP5 knockdown (Fig 5E). Given that siFABP5#2 exhibited more pronounced tumor-suppressive effects in the CCK-8, colony formation, scratch wound, and EdU assays, we further analyzed proliferation- and migration-related protein expression in Huh7-siFABP5#2 cells. The results showed that knockdown of FABP5 notably up-regulated the epithelial marker E-cadherin, while down-regulating the mesenchymal markers N-cadherin and Vimentin (Fig 5F), suggesting suppression of the epithelial-mesenchymal transition (EMT) process. Taken together, these results indicate that FABP5 knockdown suppresses hepatocellular carcinoma cell proliferation *in vitro*.

**Fig 5:**
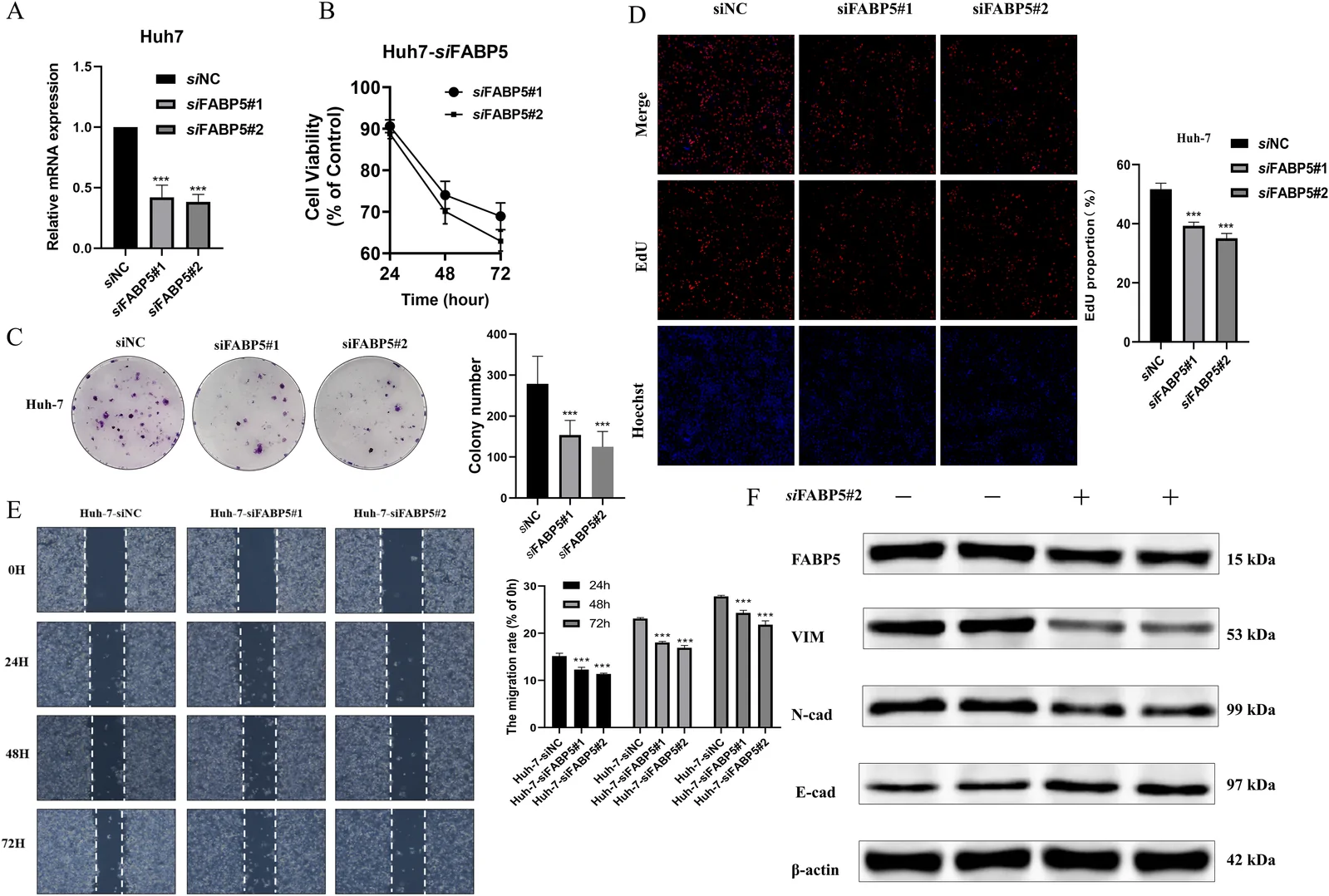
FABP5 knockdown suppresses HCC cell proliferation *in vitro.* (A) qPCR analysis confirmed effective silencing of FABP5 by siRNA in Huh7 cells. (B) CCK-8 assay showed that FABP5 knockdown reduced cell viability at 24, 48, and 72 hours. (C) Colony formation assay demonstrated significantly fewer colonies after FABP5 knockdown. (D) EdU assay confirmed reduced proliferative capacity in FABP5- knockdown Huh7 cells. (E) The wound assay showed the healing capability of Huh7 cells was markedly suppressed after FABP5 knockdown. (F) Western blot analysis demonstrated that FABP5 knockdown upregulated E-cadherin and downregulated N-cadherin and Vimentin.

### 3.6. FABP5 expression correlates with immune cell infiltration and an immunosuppressive microenvironment in HCC

CIBERSORT immune infiltration analysis was performed on batch-corrected GEO hepatocellular carcinoma datasets. First, to assess batch correction efficacy, we displayed boxplots of corrected gene expression (Fig 6A) and PCA plots (Fig 6B), demonstrating that technical batch differences between samples were effectively eliminated and expression distributions from different data sources converged. Subsequently, CIBERSORT analysis based on corrected expression matrices yielded relative abundance of 22 immune cell types in each sample, with distribution displayed via barplot (Fig 6C); immune cell infiltration differences between tumor and normal samples were compared (Fig 6D) via correlation heatmap (Fig 6E). Further analysis examined correlations between FABP5 expression and immune cell abundance using Spearman correlation. Specifically, FABP5 relative abundance showed significant positive correlation with M0 macrophages (*p* = 0.004); conversely, FABP5 demonstrated significant negative correlation with M1 macrophages (*p* = 0.015). Additionally, FABP5 expression negatively correlated with naive B cell (B cells naive) infiltration proportion (*p* = 0.009) (Fig 6F). These results suggest that elevated FABP5 expression may be associated with an increased abundance of unpolarized M0 macrophages and reduced M1 macrophage and naive B cells infiltration. To assess the potential impact of FABP5 expression levels on response to immunotherapy, we utilized the TIDE (Tumor Immune Dysfunction and Exclusion) algorithm. We found that the high FABP5 expression group exhibited a significantly elevated TIDE score compared to the low expression group (*p* = 2.2 × 10 ^−8^; Fig 6G). A higher TIDE score is a well-validated computational biomarker that predicts resistance to immune checkpoint blockade therapy. This suggests that tumors with high FABP5 expression possess an immune-suppressive microenvironment conducive to immune evasion. This conclusion is further bolstered by our concomitant observation of widespread upregulation of multiple HCC immune checkpoint molecules (e.g., PD-L1, CTLA-4, PD-1) in the same patient cohort (Fig 6H). We next calculated the statistical difference in ESTIMATE scores between the high and low FABP5 expression groups. We found significantly higher stromal, immune, and ESTIMATE scores in the high FABP5 expression group compared to the low expression group (*p* < 0.001, Fig 6I). A high immune score typically indicates lower tumor purity and the presence of greater immune cell infiltration within the tumor microenvironment, which may be associated with either immune activation or immune escape.

**Fig 6:**
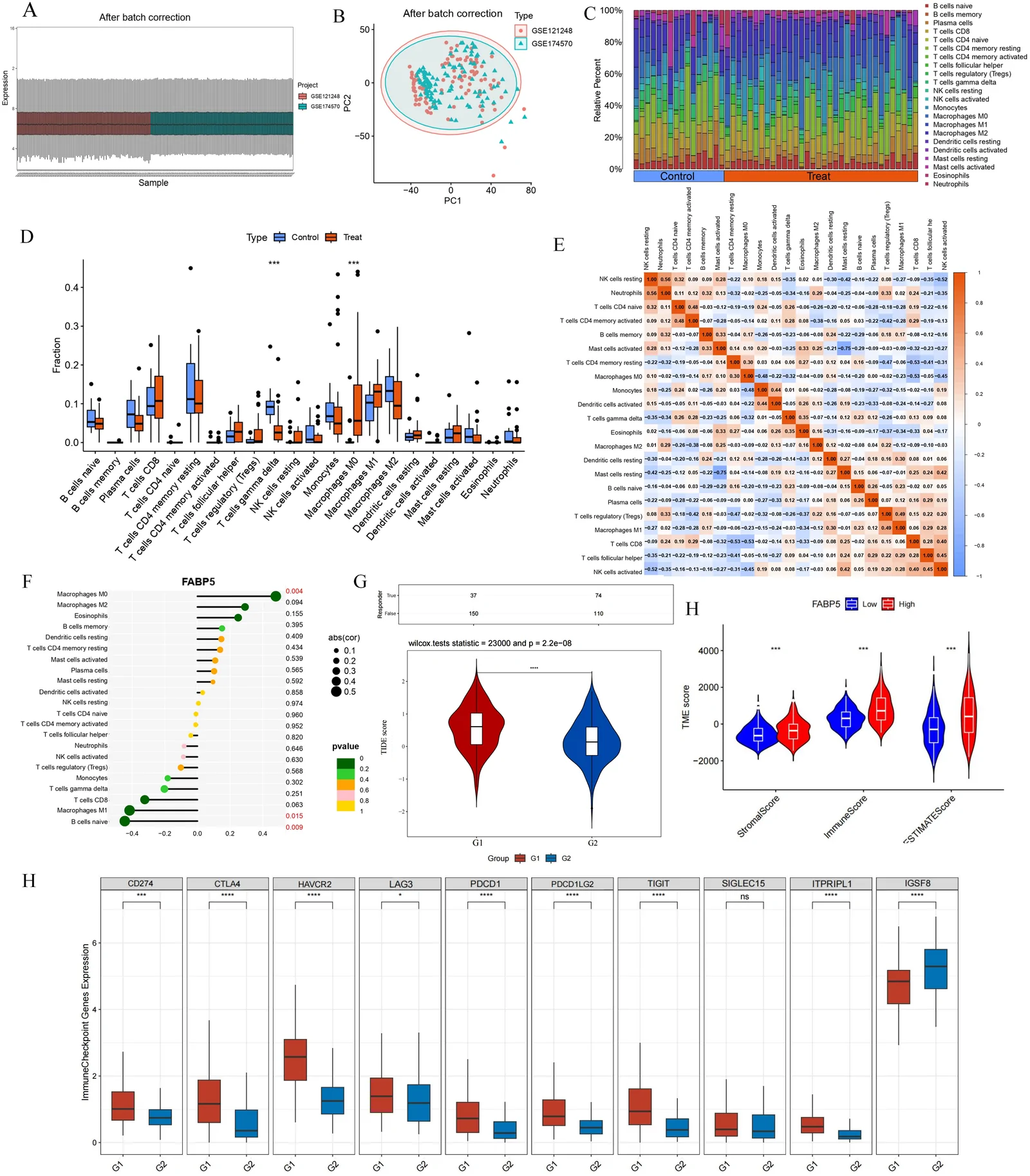
FABP5 expression correlates with immune infiltration and immunosuppressive microenvironment. (A) Boxplots of normalized gene expression after batch correction. (B) PCA plots demonstrating the effectiveness of batch effect removal. (C) Relative abundance of 22 immune cell subsets estimated by CIBERSORT. (D) Differential infiltration analysis of immune cell populations between tumor and normal tissues. (E) Correlation matrix of infiltrating immune cells. (F) Correlation analysis between FABP5 expression and immune cell abundance. (G) TIDE analysis showing higher TIDE scores in FABP5-high patients, suggesting resistance to immune checkpoint blockade therapy. (H) Upregulation of multiple immune checkpoint molecules (e.g., PD-L1, CTLA-4, PD-1) in FABP5-high tumors. (I) ESTIMATE analysis confirmed higher stromal and immune scores in the FABP5-high group. G1: Group 1 (High Expression); G2: Group 2 (Low Expression). *p < 0.05, **p < 0.01, ***p < 0.001.

### 3.7. Functional enrichment analysis links FABP5 to immune activation and NF-κB signaling pathways

To investigate the specific mechanisms by which FABP5 mediates tumor immune infiltration, we examined the differentially expressed genes (DEGs) between the low- and high-expression groups (Fig 7A). Enriched biological process (BP) terms were associated with immunity, including “leukocyte mediated immunity”, “lymphocyte mediated immunity”, “adaptive immune response based on somatic recombination of immune receptors built from immunoglobulin superfamily domains”, “B cell mediated immunity”, “immunoglobulin mediated immune response”, and “immune response-regulating cell surface receptor signaling pathway”. The enriched cellular component (CC) terms were related to “immunoglobulin complex”, “T cell receptor complex”, “plasma membrane signaling receptor complex”, “external side of plasma membrane”, “IgG immunoglobulin complex”, and “tertiary granule membrane”. For molecular function (MF), DEGs were mainly enriched in “antigen binding”, “immune receptor activity”, “peptide antigen binding”, “carbohydrate binding”, “cytokine receptor activity”, and “cytokine activity” (Fig 7B). KEGG pathway enrichment analysis revealed that high FABP5 expression was related to the “hsa04060 Cytokine-cytokine receptor interaction”, “hsa04062 Chemokine signaling pathway”, “hsa04658 Th1 and Th2 cell differentiation”, “hsa04659 Th17 cell differentiation”, “hsa04662 B cell receptor signaling pathway”, “hsa04064 NF‑κB signaling pathway”, “hsa04151 PI3K‑Akt signaling pathway”, “hsa04660 T cell receptor signaling pathway”, “hsa04657 IL-17 signaling pathway”, and “hsa04630 JAK-STAT signaling pathway” (Fig 7C). These results indicate that FABP5 might influence tumor immune infiltration through multiple pathways.

**Fig 7:**
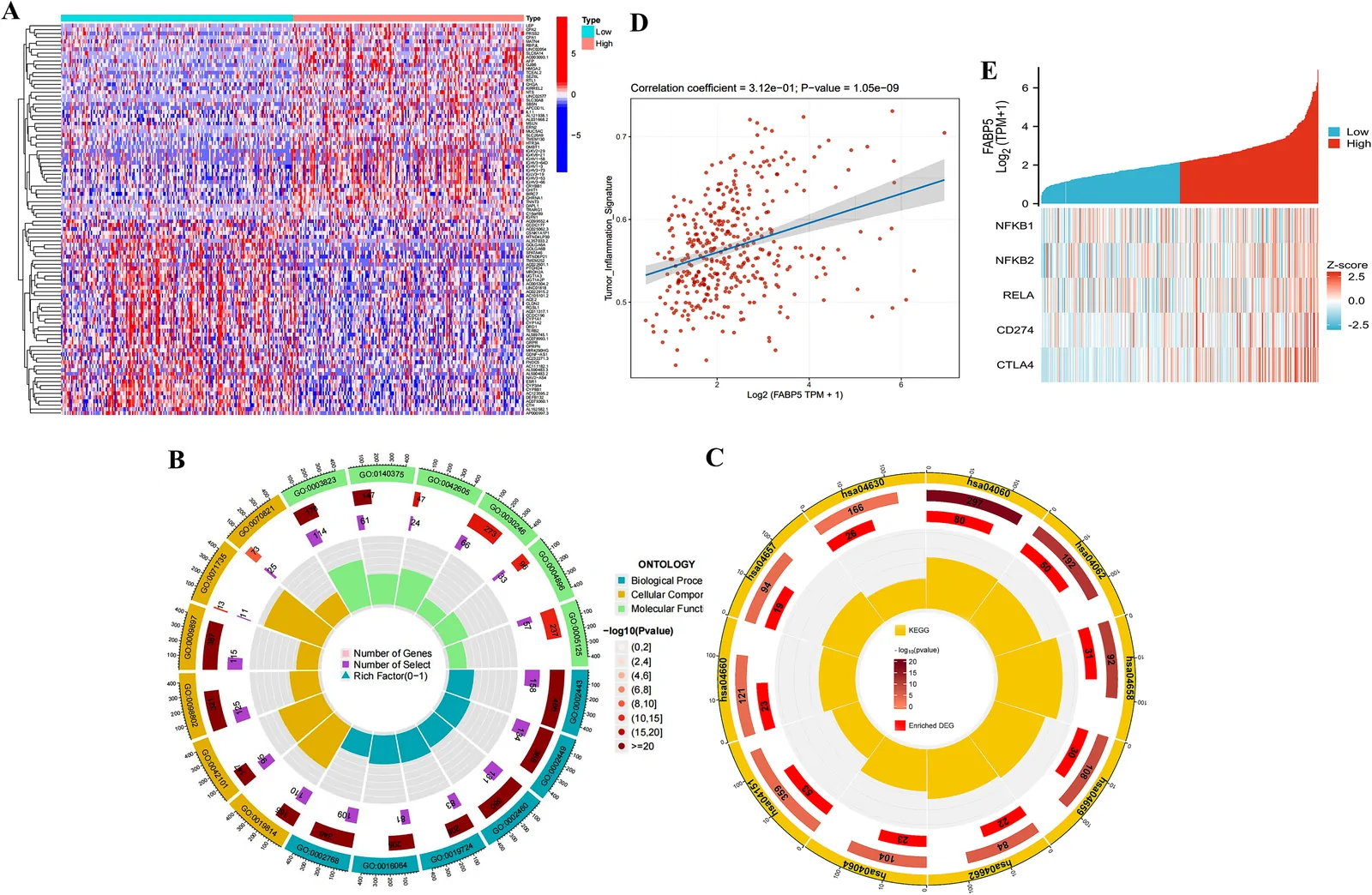
Functional enrichment analysis links FABP5 to immune activation and NF-κB signaling pathways. (A) Heatmap of DEGs between high and low FABP5 expression groups. (B) Gene Ontology (GO) enrichment analysis of DEGs, showing significant biological processes (BP), cellular components (CC), and molecular functions (MF) related to immune regulation. (C) KEGG pathway enrichment analysis highlighting immune-related pathways associated with FABP5 expression, such as cytokine–cytokine receptor interaction, chemokine signaling, Th1/Th2 and Th17 cell differentiation, B cell receptor signaling, NF-κB signaling, PI3K–Akt signaling, T cell receptor signaling, IL-17 signaling, and JAK–STAT signaling. (D) Correlation analysis revealed a positive association between FABP5 expression and cancer inflammatory gene signatures. (E) Heatmap showing co-upregulation of NF-κB pathway genes (NF-κB1, NF-κB2, RELA) in FABP5-high tumors.

To further investigate the relationship between FABP5 and the NF-κB signaling pathway, which is critically involved in tumor-related inflammation, we analyzed the correlation between FABP5 expression and a tumor inflammatory signature. As shown in Fig 7D, FABP5 expression exhibited a significant positive correlation with the inflammatory signature. Concurrently, the heatmap in Fig 7E demonstrated that high FABP5 expression was associated with elevated mRNA levels of key genes in the NF-κB signaling pathway, including NFKB1, NFKB2, and RELA.

### 3.8. Computational and experimental evidence supports FABP5–NF-κB axis activation

To explore potential direct molecular interactions, protein–protein docking simulations were performed between FABP5 and major components of the NF-κB pathway, including IκBα, IKKβ, and P65 (RelA). Among ten predicted docking conformations, the top-ranked complexes were visualized and analyzed. The FABP5–IκBα complex exhibited a docking score of –173.36, a confidence score of 0.6147 (Fig 8A). The FABP5–IKKβ complex achieved a docking score of –211.73 (confidence = 0.7746) (Fig 8B), while the FABP5–P65 complex displayed predicted interaction, with a docking score of –220.06, a confidence score of 0.8024 (Fig 8C). To further characterize the stability and dynamic behavior of the FABP5–NF-κB pathway protein complexes, molecular dynamics (MD) simulations were performed, and the results are summarized in Fig 8. As shown in Fig 8D, the root mean square deviation (RMSD) of the FABP5–IκBα complex reached equilibrium after approximately 80 ns and fluctuated around 8.6 Å, indicating overall structural stability. Similarly, the FABP5–P65 complex stabilized after 80 ns, with RMSD values fluctuating around 5 Å. These results suggest that both IκBα and P65 form complexes with FABP5. In contrast, the FABP5–IKKβ complex exhibited a continuous upward trend in RMSD during the simulation, suggesting notable conformational changes and reduced complex stability. Analysis of the radius of gyration (Rg) further supported these observations. As shown in Fig 8E, the FABP5–IκBα and FABP5–P65 (NET1) complexes displayed relatively stable Rg values throughout the simulation, indicating that no pronounced global expansion or contraction occurred during ligand binding, and that the overall compactness of the complexes was maintained. Solvent-accessible surface area (SASA) analysis revealed mild fluctuations in the FABP5–IκBα complex (Fig 8F), suggesting that ligand binding moderately altered the local binding microenvironment and resulted in limited changes in solvent exposure. In contrast, the SASA of the FABP5–P65 complex remained largely unchanged upon binding, indicating that ligand association exerted minimal impact on the overall protein conformation. The number of hydrogen bonds formed between the ligand and target proteins during the MD simulations is shown in Fig 8G. For the FABP5–IκBα complex, the number of hydrogen bonds ranged from 0 to 10, with approximately six hydrogen bonds maintained for most of the simulation time. For the FABP5–P65 complex, the number of hydrogen bonds ranged from 0 to 19, with an average of approximately eleven hydrogen bonds, indicating hydrogen-bonding interactions that contribute to complex stability. Root mean square fluctuation (RMSF) analysis further demonstrated that both complexes exhibited limited residue-level flexibility. As shown in Fig 8H, RMSF values for the FABP5–IκBα complex were mostly below 6 Å, while those for the FABP5–P65 complex were largely below 4 Å, indicating relatively low flexibility and high structural stability. The hydrogen bonds and RMSF analysis of FABP5–IKKβ complex were shown in S1A and S1B Figs. Binding free energies were subsequently calculated using the MM/PBSA method based on representative complex conformations (S1C Fig). The binding free energies of the FABP5–IKKβ, FABP5–IκBα, and FABP5–P65 complexes were −72.39 kcal/mol, −7.76 kcal/mol, and −18.43 kcal/mol, respectively, indicating favorable binding affinity, with lower energy values suggesting potentially stronger interactions. To further identify residues contributing critically to ligand binding, per-residue free energy decomposition was performed (Fig 8I and S1D Fig). In the FABP5–IκBα complex, residues ILE65, TYR84, VAL81, LEU62, VAL61, VAL88, and TYR19 exhibited high contribution values. In the FABP5–P65 (NET1) complex, residues ARG198, ARG41, ARG35, LYS218, and PHE213 showed relatively higher contributions. These findings suggest that these amino acid residues play important roles in stabilizing the FABP5–NF-κB protein complexes. Collectively, these in silico results suggest that FABP5 may form complexes with IκBα and P65 (NET1) under the conditions tested, with predicted binding stability and hydrogen-bonding interactions. However, it remains necessary to confirm through direct experimental validation (e.g., co-immunoprecipitation, pull‑down assays or confocal colocalization assays) in future studies whether these predicted interactions are indeed present in HCC cells.

**Fig 8:**
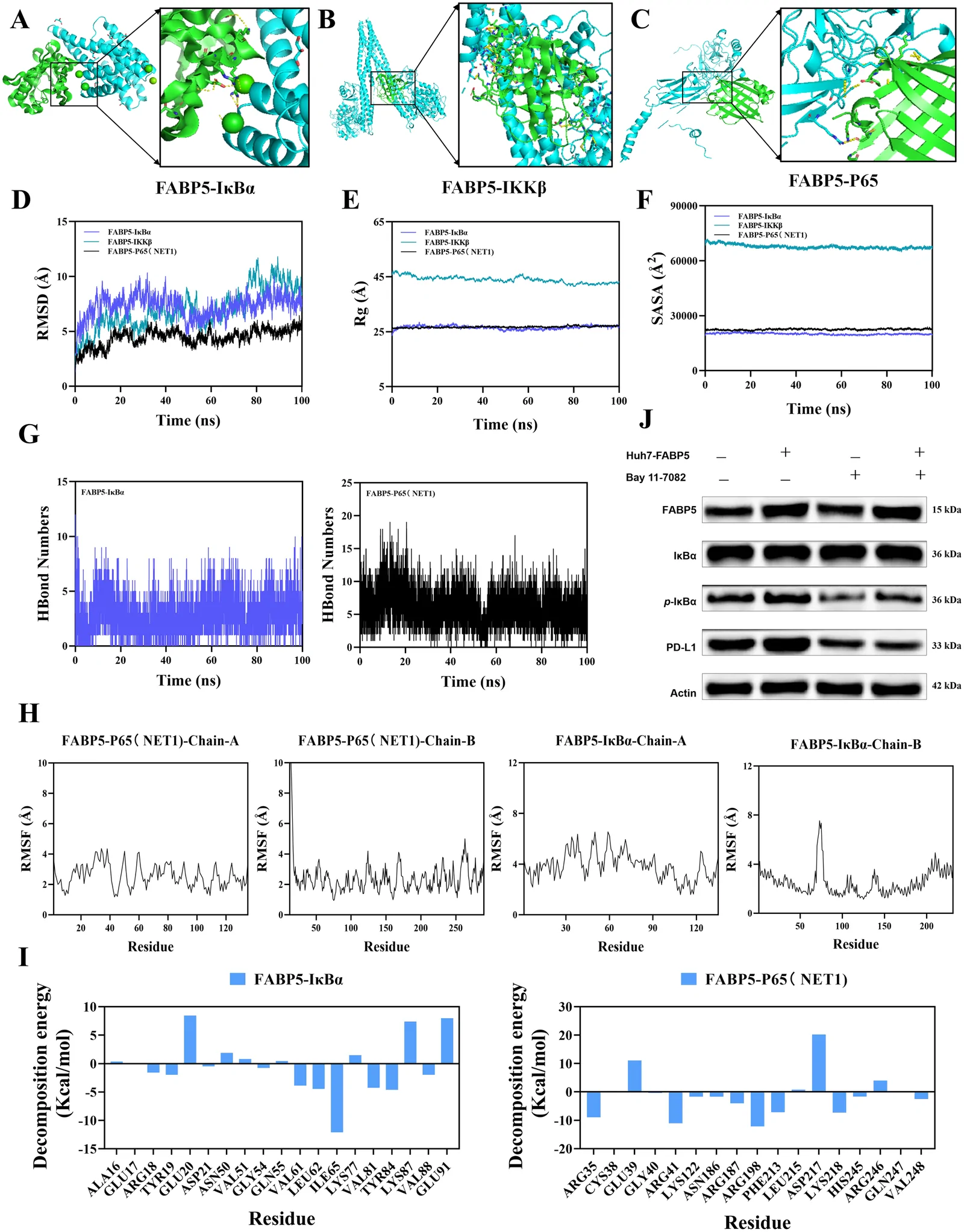
Computational and experimental evidence supporting activation of the FABP5–NF-κB signaling axis. (A–C) Representative top-ranked protein–protein docking conformations of FABP5 with IκBα (A), IKKβ (B), and P65 (RelA) (C). (D) RMSD profiles from MD simulations showing that the FABP5–IκBα and FABP5–P65 complexes reached equilibrium after ~80 ns, whereas the FABP5–IKKβ complex exhibited reduced stability. (E) Rg analysis demonstrating stable global compactness of the FABP5–IκBα, FABP5-IKKβ and FABP5–P65 complexes during MD simulations. (F) SASA analysis showing mild fluctuations for the FABP5–IκBα and FABP5-IKKβ complex and minimal variation for the FABP5–P65 complex. (G) Hydrogen bond analysis illustrating persistent intermolecular hydrogen bonding between FABP5 and NF-κB pathway proteins, with a higher average number observed in the FABP5–P65 complex. (H) RMSF analysis indicating limited residue-level flexibility and high structural stability of the FABP5–IκBα and FABP5–P65 complexes. (I) Residue free energy decomposition identifying key amino acid residues contributing to stabilization of the FABP5–IκBα and FABP5–P65 complexes. (J) Western blot analysis showing that FABP5 overexpression increased phosphorylation of IκBα (p-IκBα) and PD-L1 expression in HCC cells, while NF-κB inhibition by Bay 11–7082 abolished these effects.

To establish a functional link, HCC cells overexpressing FABP5 were treated with the NF-κB pathway inhibitor Bay 11‑7082. Phosphorylated IκBα (p-IκBα) was used as a readout of NF-κB activation. Western blotting showed that FABP5 overexpression increased p-IκBα and PD-L1 expression, whereas co-treatment with the NF-κB inhibitor Bay 11‑7082 abolished these increases, returning PD-L1 levels to baseline. These results suggest that the ability of FABP5 to upregulate PD-L1 requires NF-κB signaling activity, indicating that FABP5 acts, at least in part, through the NF-κB pathway.

### 3.9. CZS-241 as a promising FABP5-targeting agent in HCC: insights from molecular docking analysis

Given the significant effect of FABP5 gene knockdown in suppressing HCC cell proliferation, we screened out 5 potential drugs targeting FABP5 through the DGIdb database, namely XMD8–92, OCIFISERTIB, AXITINIB, RG-1530 and CZS-241. Molecular docking analysis indicated that the binding energies of XMD8–92, OCIFISERTIB, AXITINIB, RG-1530 and CZS-241 were −7.9 kcal/mol, −10.7 kcal/mol, −9 kcal/mol, −8.6 kcal/mol and −11 kcal/mol, respectively (Figs 9A–9E). The above results suggest that CZS-241 might have potential strong binding interactions with FABP5.

**Fig 9:**
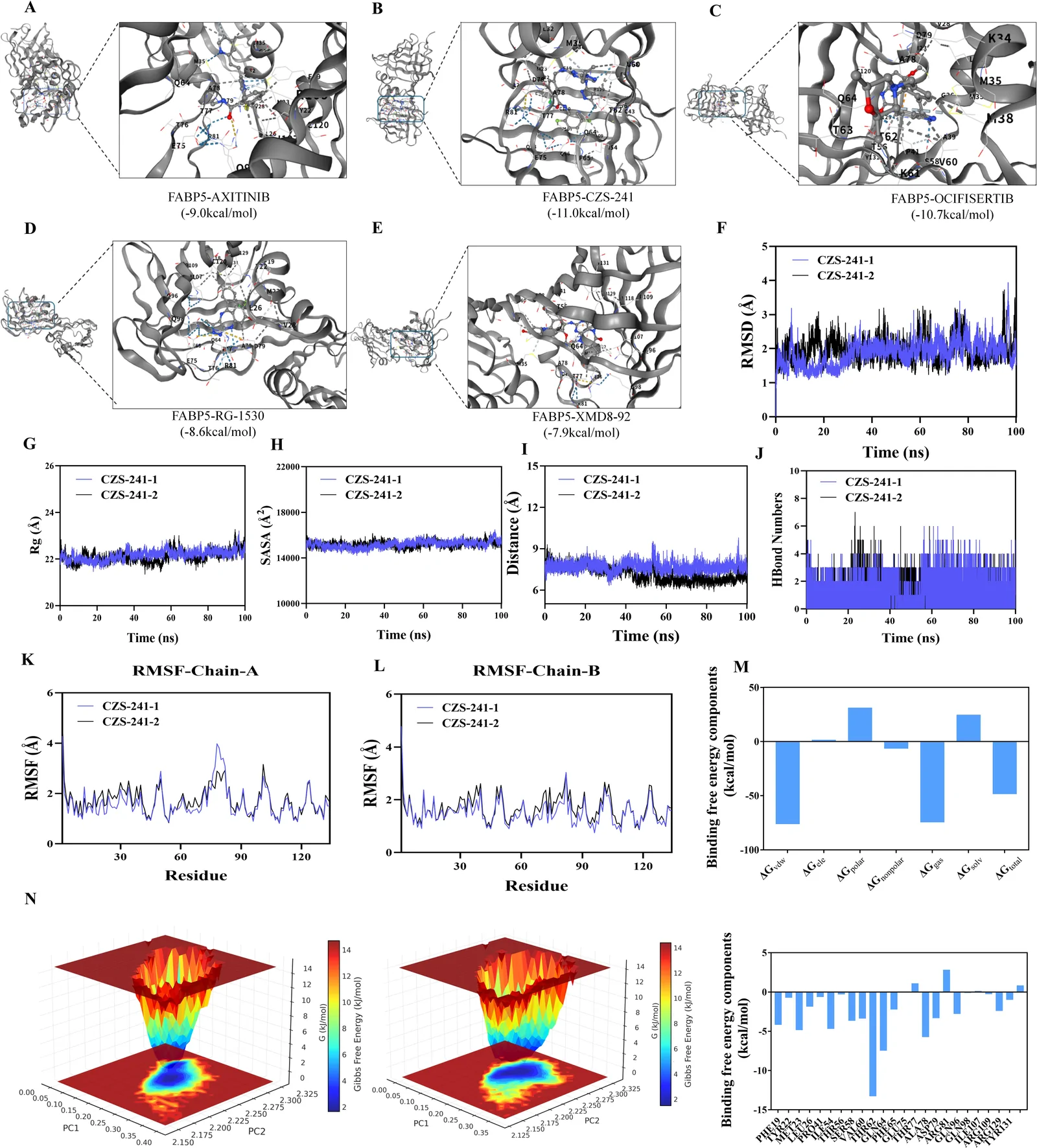
Reproducible molecular dynamics simulations and binding free energy analysis of the CZS-241–FABP5. (A-E)Molecular docking analysis between FABP5 and 5 potential drugs including XMD8–92, OCIFISERTIB, AXITINIB, RG-1530 and CZS-241. (F) RMSD profiles from duplicate molecular dynamics (MD) simulations showing that the CZS-241–FABP5 complex remained stable with fluctuations below 3 Å throughout the simulation. (G) Rg analysis indicating stable global compactness of the CZS-241–FABP5 complex with only minor conformational fluctuations during MD simulations. (H) SASA analysis showing slight variations, suggesting limited changes in the binding microenvironment upon ligand association. (I) Time-dependent evolution of the distance between CZS-241 and the key interacting residue THR56, illustrating transient fluctuations followed by convergence to a stable binding distance. (J) Hydrogen bond analysis demonstrating persistent intermolecular hydrogen bonding between CZS-241 and FABP5, with an average of approximately three hydrogen bonds maintained during the simulation.(K–L) RMSF profiles indicating low residue-level flexibility (<3 Å for most residues), consistent with a stable ligand–protein complex. (M) MM/PBSA binding free energy analysis showing a favorable average binding free energy (−48.48 kcal/mol) for the CZS-241–FABP5 complex, with per-residue energy decomposition identifying key amino acid residues contributing to ligand binding. (N) Free energy landscape (FEL) analyses of the FABP5–CZS-241 complex from two independent molecular dynamics simulations. The left panel represents replicate 1 and the right panel represents replicate 2.

### 3.10. Duplicate molecular dynamics simulations confirm the stable binding of CZS-241 to FABP5

Duplicate molecular dynamics simulations were performed with different initial random velocities to ensure reproducibility. As shown in Fig 9F, the CZS-241–FABP5 complex exhibited only minor fluctuations in RMSD values below 3 Å throughout the simulation, indicating a low overall deviation and high structural stability of the ligand–protein complex. Further analyses demonstrated that both the Rg and the SASA of the CZS-241 complex displayed only slight fluctuations during the simulation (Figs 9G and 9H), suggesting that the ligand–protein complex underwent minor conformational adjustments while maintaining an overall stable structural framework. The evolution of key residue distances reflects the temporal changes in distances between specific ligand atoms and interacting residues within the protein. We selected amino acid residues that formed hydrogen bonds with the ligand in the molecular docking results for distance evolution analysis. As shown in Fig 9I, the distance between the ligand and the key residue THR56 exhibited certain fluctuations during the simulation; however, this distance gradually stabilized toward the end of the trajectory. These results further indicate that although limited conformational rearrangements occurred during the simulation, the complex ultimately reached a stable binding state. Hydrogen bonds play a critical role in stabilizing ligand–protein interactions. As illustrated in Fig 9J, the number of hydrogen bonds formed between CZS-241 and FABP5 during the simulation ranged from 0 to 7, with approximately three hydrogen bonds maintained for most of the simulation time. This observation suggests the presence of persistent and favorable hydrogen-bonding interactions between the ligand and the target protein. Consistent with this, the RMSF values of the complex were relatively low, with most residues fluctuating below 3 Å (Figs 9K and 9L), indicating limited residue flexibility and high structural stability of the complex. Subsequently, binding free energy calculations were performed using the MM/PBSA method based on the representative binding conformations extracted from the simulation trajectories (Fig 9M). The average binding free energy of the CZS-241–FABP5 complex was calculated to be −48.48 kcal/mol. To further identify residues contributing significantly to ligand binding, per-residue free energy decomposition analysis was conducted. The results revealed that residues VAL60, THR77, THR56, PRO41, SER58, PHE19, and MET23 exhibited high energetic contributions in the CZS-241–FABP5 complex, suggesting that these amino acid residues may play critical roles in stabilizing the ligand–protein interaction. To further characterize the conformational stability of the FABP5–CZS-241 complex, free energy landscape (FEL) analyses were performed based on the molecular dynamics trajectories. As shown in Fig 9N (left, replicate 1; right, replicate 2), both simulations exhibited a predominant low-energy basin centered around RMSD ≈ 0.18 nm and Rg ≈ 2.25–2.26 nm. The presence of a well-defined low-energy basin suggests that the complex converged toward a thermodynamically favorable conformational state during the simulations, further supporting the stability of the FABP5–CZS-241 interaction. Collectively, these results demonstrate that the CZS-241–FABP5 complex exhibits high binding stability and robust hydrogen-bonding interactions, supporting the conclusion that the small-molecule compound CZS-241 binds favorably and stably to the target protein FABP5.

## 4. Discussion

The study positions FABP5 as a pivotal oncogenic driver and a key regulator of the immunosuppressive tumor microenvironment (TIME) in HCC. We systematically demonstrated that FABP5 is significantly overexpressed in HCC tissues and cell lines, and that its elevated expression is strongly associated with adverse clinicopathological features and serves as an independent prognostic factor. Functional assays validated its essential role in promoting HCC cell proliferation in vitro*.* Mechanistically, using an integrated multi-omics approach, we uncovered a paradoxical role of FABP5 in orchestrating TIME remodeling. High FABP5 expression correlated with enhanced infiltration of diverse immune cell types, including CD8 ⁺ T cells and macrophages. However, this was accompanied by an immunosuppressive landscape characterized by the universal upregulation of immune checkpoint molecules (e.g., PD-L1, CTLA-4, PD-1) and a significantly elevated Tumor Immune Dysfunction and Exclusion (TIDE) score, predicting a non-responsive phenotype to immune checkpoint blockade therapy [13]. Single-cell transcriptomic profiling identified fibroblasts and macrophages as primary cellular sources of FABP5 within the TIME, suggesting its role in mediating stromal-immune crosstalk. Functional enrichment analyses further implicated FABP5 in critical immune-related pathways notably the NF-κB signaling pathway. In vitro functional assays demonstrated its capacity to upregulate PD-L1 expression via the NF-κB pathway. Molecular docking and molecular dynamics simulations were performed to explore potential therapeutic inhibitors of FABP5. Docking analysis identified CZS-241 as the most promising candidate, and subsequent molecular dynamics simulations validated the stability of the CZS-241–FABP5 complex. Collectively, our findings position FABP5 not only as a prognostic biomarker but also as a player in immune evasion, highlighting its dual potential as a therapeutic target for both tumor cells and the immunosuppressive microenvironment. Furthermore, we provide dynamic evidence that CZS-241 may serve as a potent FABP5 inhibitor, offering a mechanistic rationale for therapeutic targeting in HCC.

We found that high FABP5 expression is associated with a significant increase in the infiltration of various immune cells, including CD8 ⁺ T cells and macrophages. However, this influx is paradoxically accompanied by marked immunosuppression and poorer patient outcomes. This phenomenon can be explained by the fact that although immune cells are effectively recruited to the tumor site, they become functionally inert or exhausted, being trapped within a suppressive tumor microenvironment (TME) that prevents effective antitumor immunity. A key mechanism by which the TME suppresses immune function is the upregulation of inhibitory receptors on T cells. For instance, the expression of PD-1, along with other inhibitory receptors such as T-cell immunoglobulin and mucin domain-containing protein 3 (TIM-3) and CTLA-4, on tumor-infiltrating lymphocytes (TILs) represents a hallmark of T-cell exhaustion [15–17]. Consistently, our data demonstrate that in HCC, patients with high FABP5 expression exhibit increased expression of immune checkpoint genes, including PD-1 and CTLA-4. Therefore, FABP5 may serve not only as a tumor biomarker but also as an active regulator of the immunosuppressive TME. The mechanisms underlying this phenomenon are multifaceted, which may relate to FABP5’s fundamental role as a lipid chaperone. FABP5-mediated lipid signaling has been implicated in reprogramming of the TME, fostering an immunosuppressive environment that supports tumor growth and metastasis. One of the key mechanisms by which FABP5 contributes to immunosuppression is through its interaction with tumor-associated macrophages (TAMs). Studies have shown that FABP5 activation in TAMs leads to the accumulation of lipid droplets and the suppression of fatty acid β-oxidation, which in turn promotes the expression of immunosuppressive molecules such as PD-L1 on regulatory T cells (Tregs) via the JNK-STAT3 pathway [18]. This lipid accumulation and metabolic reprogramming of TAMs are crucial for maintaining an immunosuppressive TME, thereby facilitating tumor progression and reducing the efficacy of immune checkpoint blockade therapies [19]. Furthermore, FABP5’s role in lipid metabolism extends to its influence on T cell function within the TME. FABP5 expression in T cells has been shown to regulate fatty acid uptake and oxidation, which are critical for the survival and function of different T cell subsets in the nutrient-deprived TME [20]. This regulation is particularly important, as T cells often exhibit an exhausted phenotype in the TME due to limited glucose availability, and FABP5-mediated lipid metabolism may provide an alternative energy source that supports T cell function and anti-tumor immunity. Thus, FABP5 plays a pivotal role in reprogramming the TME to support immunosuppression through its regulation of lipid metabolism in both TAMs and T cells.

Beyond metabolic reprogramming, our pathway enrichment analyses revealed the activation of critical signaling networks that propagate a chronic inflammatory state. The significant enrichment of pathways including “Cytokine-cytokine receptor interaction,” “NF-kappa B signaling,” and “JAK-STAT signaling” provides a transcriptional framework for FABP5’s actions. As a nuclear transcription factor, NF-κB plays a pivotal role in regulating both inflammatory and immune responses. In nasopharyngeal carcinoma, the interaction between NF-κB and the STAT3 signaling pathway has been shown to upregulate PD-L1 expression on vascular endothelial cells, thereby promoting immune evasion [21]. Similar mechanisms have been observed in other cancer types. For instance, in diffuse large B-cell lymphoma, LMP2A encoded by Epstein–Barr virus enhances immune evasion through the upregulation of the SYK/NF-κB signaling pathway [22]. Both NF-κB and JAK-STAT signaling are powerful inducers of immune checkpoint molecules. Furthermore, the NF-κB signaling pathway plays a significant role in the TME. Studies indicate that NF-κB serves as a central component in the TME of osteosarcoma, influencing the recruitment and polarization of tumor-associated macrophages (TAMs) and myeloid-derived suppressor cells (MDSCs), thereby promoting immune suppression [23]. In colorectal cancer, NF-κB regulates the expression of inflammatory factors such as TNF, IL-1β, and IL-6, which facilitate EMT and enhance cancer invasion and metastasis [24]. Notably, the interaction between NF-κB and other signaling pathways plays a crucial role in cancer immune evasion. Research has revealed complex crosstalk between the Wnt/β-catenin signaling pathway and NF-κB, which significantly contributes to inflammatory processes and inflammation-related diseases such as cancer [25]. Additionally, the molecular interaction between NR4A orphan nuclear receptors and NF-κB serves as a key regulator in maintaining immune cell homeostasis and modulating inflammatory responses [26]. Based on the results of KEGG and GO enrichment analyses, we propose that FABP5 may contribute to the establishment of a sustained chronic inflammatory microenvironment through the activation of these canonical inflammatory and immunoregulatory pathways. This mechanism provides a plausible explanation for the observed upregulation of immune checkpoint molecules, including PD-L1 and CTLA-4, in FABP5-high tumors. To experimentally validate this link in HCC, we treated FABP5-knockdown cells with an NF-κB inhibitor. The results demonstrated that FABP5-mediated upregulation of PD-L1 is mediated through the NF-κB pathway. This offers a direct mechanistic link, explaining the coordinated upregulation of PD-L1 checkpoints observed in FABP5-high tumors, which effectively creates a barrier that shields cancer cells from immune attack.

Critically, our single-cell RNA sequencing data provide essential spatial context, revealing that the highest expression of FABP5 is localized not within the malignant cells themselves but predominantly within the stromal compartment, specifically in cancer-associated fibroblasts (CAFs) and macrophages. CAFs and TAMs are pivotal components of TME that significantly influence cancer progression and the immune landscape within tumors. CAFs, through their diverse roles, contribute to the immunosuppressive milieu of the TME by secreting cytokines and chemokines that modulate immune cell recruitment and function. This immunosuppressive environment is further compounded by the presence of TAMs, particularly those polarized toward an M2-like phenotype, which are known for their pro-tumorigenic and immunosuppressive activities [27,28]. The interplay between CAFs and TAMs is complex and involves reciprocal interactions that enhance tumor progression and immune evasion [29]. The interaction between CAFs and TAMs is particularly noteworthy in the context of immunoregulation. CAFs have been shown to recruit monocytes and promote their differentiation into M2-like macrophages, which further contribute to the immunosuppressive TME by inhibiting T cell function and promoting tumor cell invasion and metastasis [15,30]. This recruitment and polarization of macrophages by CAFs is mediated through various signaling pathways, including the secretion of cytokines such as IL-8 and the expression of surface molecules such as VCAM-1 [31,32]. The synergistic relationship between CAFs and TAMs highlights the potential for therapeutic strategies that target both cell types to disrupt their pro-tumorigenic interactions and enhance the efficacy of immunotherapies [29,33]. The single-cell RNA sequencing data shows FABP5 is highly expressed in CAFs and macrophages. This cellular distribution pattern suggests that FABP5 may influence tumor progression by regulating immune and stromal cell functions within the microenvironment. To further validate this hypothesis, we utilized bulk transcriptomic data from GEO hepatocellular carcinoma and performed CIBERSORT immune infiltration analysis following batch effect correction. Results demonstrated that FABP5 expression levels showed significant positive correlation with M0 macrophage abundance, while demonstrating significant negative correlation with M1 macrophage and naive B cell (B cells naive) abundance. M0 macrophages exist in an unpolarized state and can be induced toward M2 phenotype or retain immunosuppressive characteristics within tumor microenvironments; conversely, M1 macrophages possess anti-tumor activity. Therefore, elevated FABP5 expression may promote tumor-associated macrophage shift toward immunosuppressive phenotypes while suppressing recruitment or differentiation of adaptive immune cells, synergistically constructing an immunosuppressive microenvironment.

To extend our functional observations, molecular docking and MD simulations were performed to further validate the interaction between FABP5 and its potential inhibitors. The docking results demonstrated that CZS-241 exhibited the most favorable binding affinity among the screened compounds, with a binding energy of –11.0 kcal/mol, suggesting strong intermolecular interactions with FABP5. To complement the docking findings, MD simulations were conducted to assess the stability of the CZS-241–FABP5 complex. RMSD and Rg analyses indicated that the complex reached equilibrium and maintained global structural stability throughout the 100 ns simulation. Furthermore, the stable hydrogen bond network within the binding pocket highlighted the stabilizing effect of CZS-241 binding on the local conformational dynamics of FABP5. Binding free energy analysis further confirmed the presence of a predominant low-energy basin, supporting the notion that the complex favors a single stable binding conformation. Collectively, these results strengthen the reliability of our docking predictions and provide dynamic insights into the molecular basis by which CZS-241 may function as a potent FABP5 inhibitor in HCC.

This study distinguishes itself through its systematic and multi-faceted approach, providing a comprehensive view of FABP5’s role in HCC. Unlike previous investigations which predominantly focused on the metabolic functions of FABP5 in lipid transport and steatotic liver disease, our work systematically elucidates its central role in immune regulation within the HCC tumor microenvironment. Our research design commenced with a pan-cancer analysis to establish the cancer type-specific significance of FABP5, followed by a focused in-depth analysis of LIHC. The immunomodulatory capacity of FABP5 offers a novel and crucial perspective for understanding the pathogenesis of MASLD-associated HCC, suggesting that immune evasion driven by metabolic dysregulation and activation of inflammatory signals may serve as a key underlying mechanism linking steatosis to carcinogenesis.

Several limitations of this study warrant acknowledgment. First, the mechanistic investigations, while suggestive, rely heavily on bioinformatic inferences and in vitro cell line models. The absence of in vivo validation using genetically engineered animal models, such as a hepatocyte-specific FABP5 knockout mouse model, limits our ability to fully ascertain the causal role of FABP5 in tumor progression and immune modulation within a physiologically intact TME. Second, the present study identified a close association between FABP5 expression and macrophage infiltration using single-cell and immune deconvolution analyses. Although we have preliminarily demonstrated by cellular experiments that FABP5 upregulates PD-L1 expression through the NF-κB pathway, the lack of deeper functional assays to directly demonstrate how FABP5 expression in tumor or stromal cells dictates immune cell function represents a significant gap. Future studies employing tumor–stromal co-culture models will be necessary to further elucidate its role in regulating the immunosuppressive tumor microenvironment. Third, our clinical correlation analyses are derived from retrospective public databases; therefore, the prognostic and predictive value of FABP5 requires further confirmation in prospective, well-designed clinical cohorts.

## 5. Conclusions

Our study demonstrates that FABP5 acts as a pivotal oncogenic driver in HCC, promoting tumor cell proliferation and remodeling the tumor immune microenvironment into an immunosuppressive state via activating the NF-κB signaling pathway. Importantly, our molecular docking and molecular dynamics simulation analyses further provide structural and energetic evidence that CZS-241 may represent a promising small-molecule inhibitor of FABP5. Its strong binding affinity and stable interaction with the FABP5 binding pocket suggest translational potential for drug development. Targeting the FABP5/NF-κB axis may suppress tumor growth and reverse immunosuppression, providing a rationale for targeted immunotherapy in HCC.

## Supporting information

S1 Raw images Original uncropped Western blot images for Figures 5−1, 5-2, 8-1, and 8-2. (PDF)

S1 Fig Molecular dynamics simulations analysis of FABP5–IKKβ complex. (A) Hydrogen bond analysis between FABP5 and IKKβ. (B) RMSF analysis of the FABP5–IKKβ complex. (C) Residue free energy decomposition identifying key amino acid residues contributing to stabilization of the FABP5–IKKβ complex. (D) Binding free energies analysis based on representative complex conformations.
(DOCX)

## Abbreviation

BP: Biological Process
CAFs: Cancer-Associated Fibroblasts
CC: Cellular Component
CCK-8: Cell Counting Kit-8
CCLE: Cancer Cell Line Encyclopedia
CI: Confidence Interval
DEGs: Differentially Expressed Genes
DMEM: Dulbecco’s Modified Eagle’s Medium
DSS: Disease-Specific Survival
EdU: 5-Ethynyl-2′-Deoxyuridine
FABP5: Fatty Acid Binding Protein 5
FBS: Fetal Bovine Serum
GO: Gene Ontology
HCC: Hepatocellular Carcinoma
HPA: Human Protein Atlas
HR: Hazard Ratio
IHC: Immunohistochemistry
KEGG: Kyoto Encyclopedia of Genes and Genomes
LGG: Lower Grade Glioma
LIHC: Liver Hepatocellular Carcinoma
LUSC: Lung Squamous Cell Carcinoma
MASLD: Metabolic Dysfunction-Associated Steatotic Liver Disease
MF: Molecular Function
NF-κB: Nuclear Factor Kappa B
OS: Overall Survival
PAAD: Pancreatic Adenocarcinoma
scRNA-seq: Single-Cell RNA Sequencing
STAD: Stomach Adenocarcinoma
TAMs: Tumor-Associated Macrophages
TCGA: The Cancer Genome Atlas
TIDE: Tumor Immune Dysfunction and Exclusion
TISCH: Tumor Immune Single-cell Hub
TME: Tumor Microenvironment
