# Supplementary material for: FABP5 regulates an immunosuppressive microenvironment in hepatocellular carcinoma through the NF-κB/PD-L1 axis
Source: PLoS One. 2026 Sep 29;21(9):e0356359. doi: 10.1371/journal.pone.0356359 (PMC13623383; doi:10.1371/journal.pone.0356359)
Supplement: Supplementary file 1 [file pone.0356359.s001.pdf]

Figure 5-1

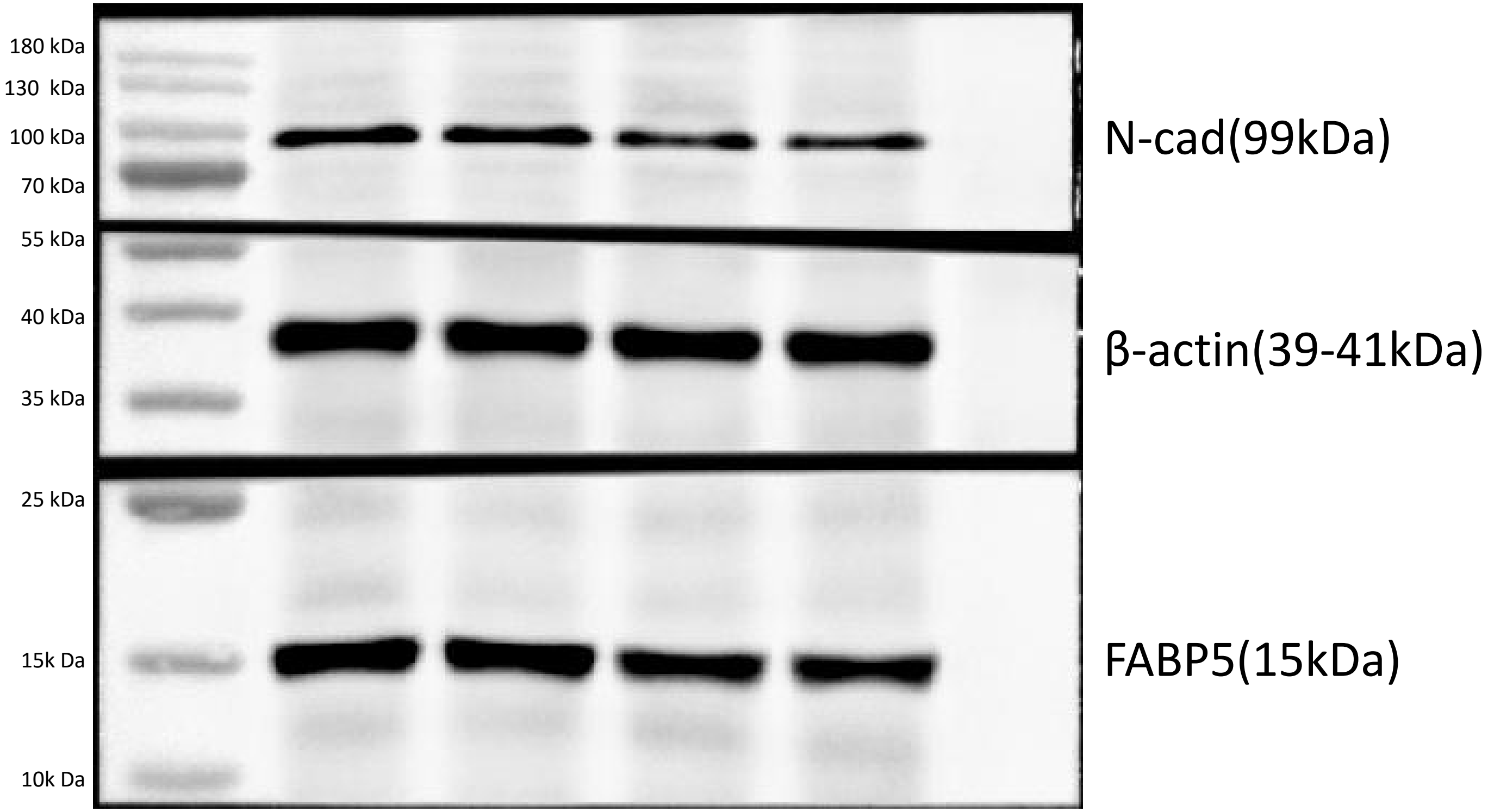
180 kDa
130 kDa
100 kDa
70 kDa
55 kDa
40 kDa
35 kDa
25 kDa
15k Da
10k Da
N-cad(99kDa)
β-actin(39-41kDa)
FABP5(15kDa)

Figure 5-2

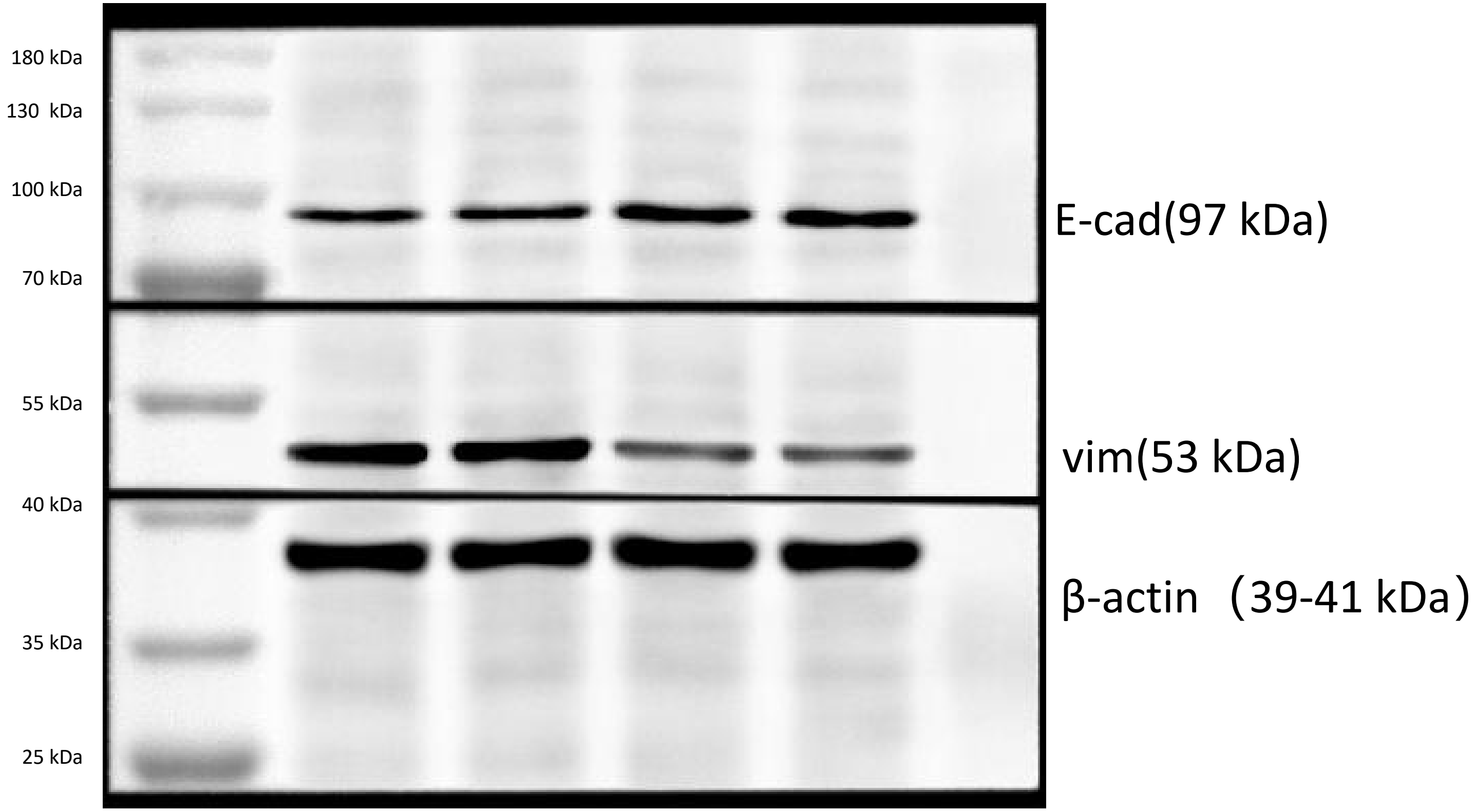
180 kDa
130 kDa
100 kDa
70 kDa
55 kDa
40 kDa
35 kDa
25 kDa
E-cad(97 kDa)
vim(53 kDa)
β-actin（39-41 kDa）

Figure 8 -1

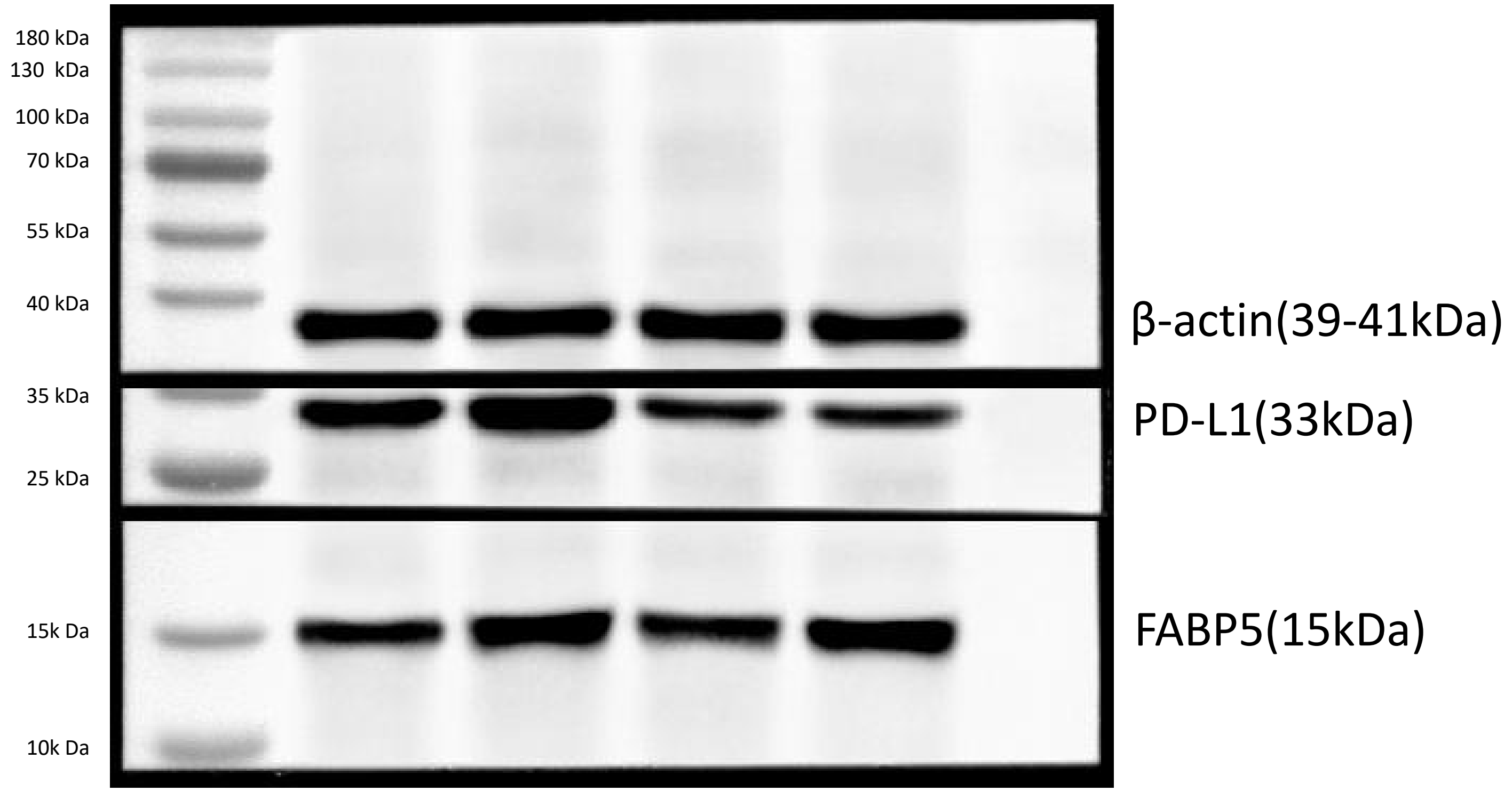
180 kDa
130 kDa
100 kDa
70 kDa
55 kDa
40 kDa
35 kDa
25 kDa
15k Da
10k Da
β-actin(39-41kDa)
PD-L1(33kDa)
FABP5(15kDa)

Figure 8 -2（1）

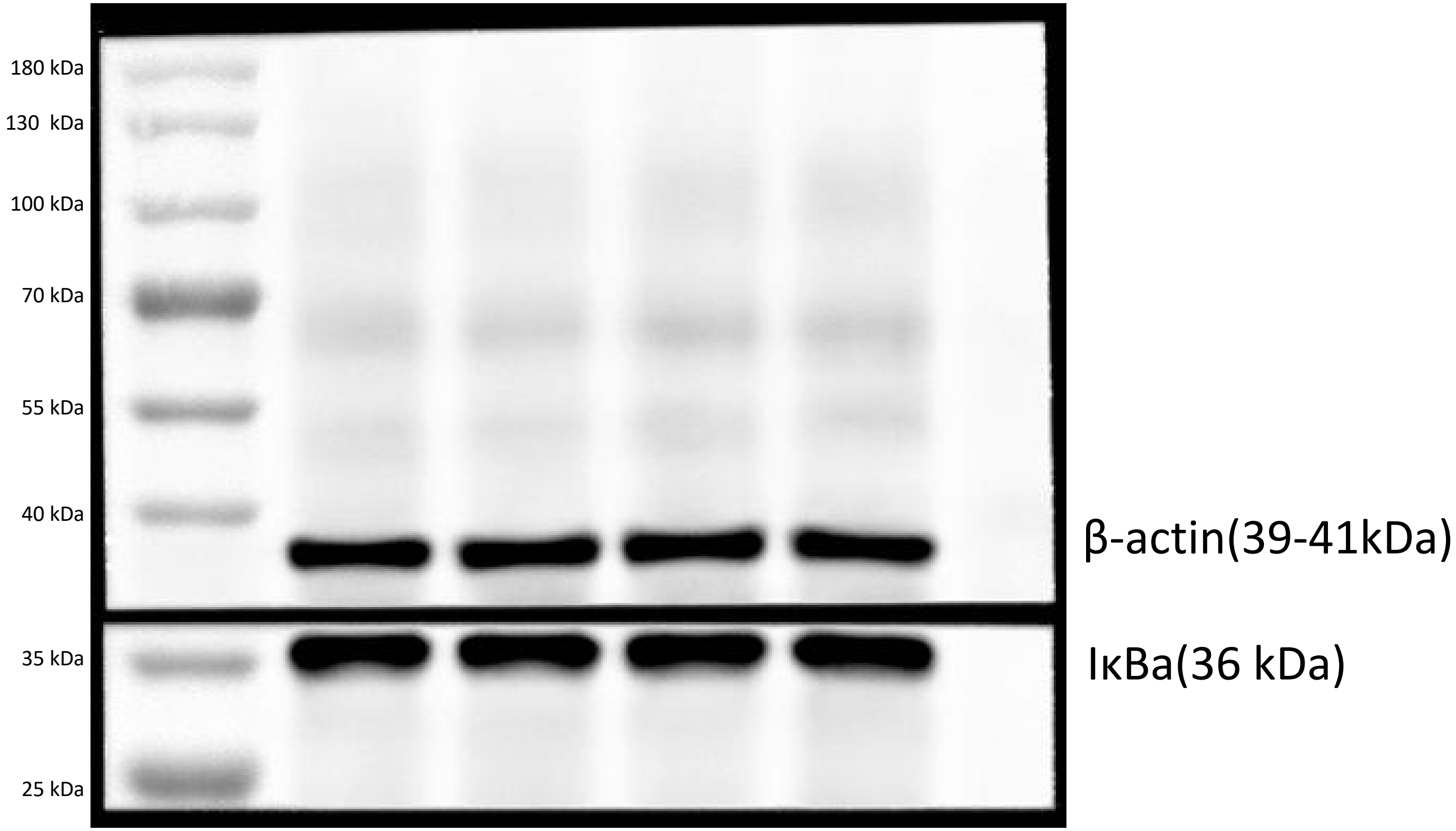
180 kDa
130 kDa
100 kDa
70 kDa
55 kDa
40 kDa
35 kDa
25 kDa
β-actin(39-41kDa)
IκBa(36 kDa)

Figure 8-2（2）

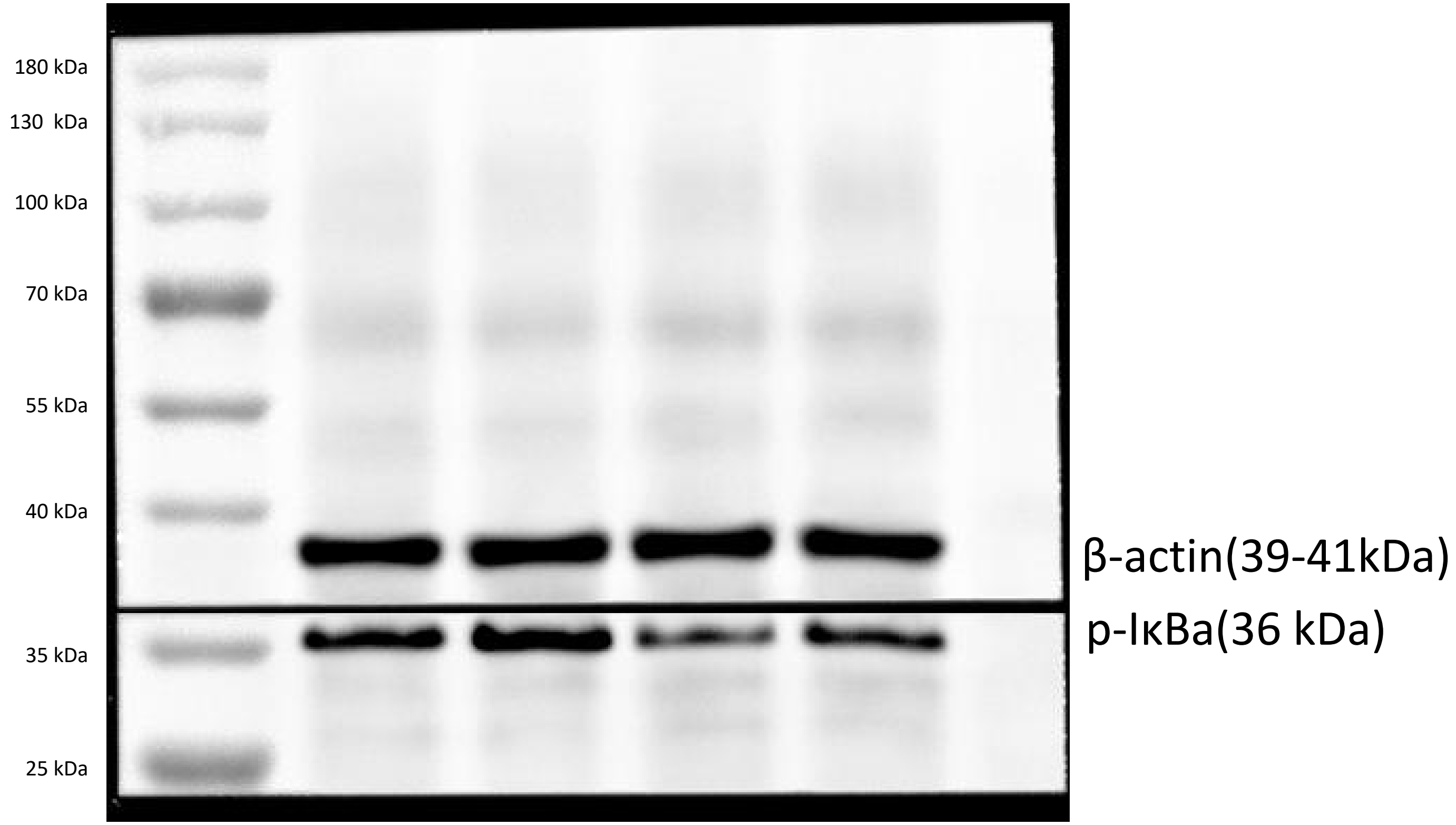
180 kDa
130 kDa
100 kDa
70 kDa
55 kDa
40 kDa
35 kDa
25 kDa
β-actin(39-41kDa)
p-IκBa(36 kDa)
