# Supplementary material for: FABP5 regulates an immunosuppressive microenvironment in hepatocellular carcinoma through the NF-κB/PD-L1 axis
Source: PLoS One. 2026 Sep 29;21(9):e0356359. doi: 10.1371/journal.pone.0356359 (PMC13623383; doi:10.1371/journal.pone.0356359)
Supplement: Supplementary file 2 [file pone.0356359.s002.docx]

S1 Fig Molecular dynamics simulations analysis of FABP5–IKKβ complex. (A) Hydrogen bond analysis between FABP5 and IKKβ. (B) RMSF analysis of the FABP5–IKKβ complex. (C) Residue free energy decomposition identifying key amino acid residues contributing to stabilization of the FABP5–IKKβ complex. (D) Binding free energies analysis based on representative complex conformations.
