# Supplementary material for: FABP5 regulates an immunosuppressive microenvironment in hepatocellular carcinoma through the NF-κB/PD-L1 axis
Source: PLoS One. 2026 Sep 29;21(9):e0356359. doi: 10.1371/journal.pone.0356359 (PMC13623383; doi:10.1371/journal.pone.0356359)
Supplement: Supplementary file 3 [file pone.0356359.s004.docx]

**Responses to Reviewers’ comments**

**Dear editor and reviewers:**

We sincerely thank you for your thoughtful comments and professional guidance. On behalf of all co-authors, I greatly appreciate the time and effort you have dedicated to our manuscript. We have carefully considered all the points raised and have revised the manuscript accordingly. Below, we provide a point-by-point response to each comment. We hope that the revised version is now suitable for publication. Thank you very much.

**Title:**FABP5 Regulates an Immunosuppressive Microenvironment in Hepatocellular Carcinoma Through the NF-κB/PD-L1 Axis.(Manuscript ID: PONE-D-26-15285 )

[Original title:FABP5 Shapes an Immunosuppressive Microenvironment in Hepatocellular Carcinoma Through the NF-κB/PD-L1 Axis.]

**-Journal Requirements**

1.Please ensure that your manuscript meets PLOS ONE’s style requirements, including those for file naming. The PLOS ONE style templates can be found at

[https://journals.plos.org/plosone/s/file?id=wjVg/PLOSOne_formatting_sample_main_body.pdf](https://journals.plos.org/plosone/s/file?id=wjVg/PLOSOne_formatting_sample_main_body.pdf) and
[https://journals.plos.org/plosone/s/file?id=ba62/PLOSOne_formatting_sample_title_authors_affiliations.pdf](https://journals.plos.org/plosone/s/file?id=ba62/PLOSOne_formatting_sample_title_authors_affiliations.pdf).

**Response:** We apologize for not adhering strictly to PLOS ONE’s style requirements in the initial submission. We have now carefully followed the guidelines and the provided templates (including the main body template and the title/authors/affiliations template) to reformat the manuscript accordingly. Specifically, we have adjusted the formatting of the title page, headings, line spacing, font, and reference style to comply with the journal’s standards. In addition, we have renamed all supplementary files according to PLOS ONE’s file naming conventions. A revised version of the manuscript with all these formatting corrections has been uploaded. We sincerely hope that the revised manuscript now meets the journal’s requirements.

2.Please note that PLOS One has specific guidelines on code sharing for submissions in which author-generated code underpins the findings in the manuscript. In these cases, we expect all author-generated code to be made available without restrictions upon publication of the work. Please review our guidelines at [https://journals.plos.org/plosone/s/materials-and-software-sharing#loc-sharing-code](https://journals.plos.org/plosone/s/materials-and-software-sharing#loc-sharing-code) and ensure that your code is shared in a way that follows best practice and facilitates reproducibility and reuse.

**Response:** We thank the Editor for highlighting the journal’s code-sharing requirements and fully recognize the importance of making computational methods openly available to ensure reproducibility and transparency.

To comply with the PLOS ONE Materials and Software Sharing Policy, we have made all author-generated code and relevant analysis outputs publicly available.

Specifically:

• The datasets used in this study were obtained from publicly accessible repositories, including The Cancer Genome Atlas (TCGA; https://portal.gdc.cancer.gov) and the Cancer Cell Line Encyclopedia (CCLE; https://depmap.org/portal/).

• The R script developed and used in this study has been deposited in the Figshare repository (https://doi.org/10.6084/m9.figshare.32415090) under the filename “FABP5_Code.R” and is freely accessible without restriction.

• The bioinformatics analysis results generated during the study have been deposited in the Figshare repository under the filename “Data for Bioinformatic Analysis.zip”.

• Molecular dynamics simulation data have also been deposited in the Figshare repository (DOI: https://doi.org/10.6084/m9.figshare.32415090) under the collection “Molecular Dynamics Simulations”.

The revised manuscript has been updated accordingly in the Data Availability Statement. We believe that the deposited code, data, and simulation files satisfy the PLOS ONE requirements for code sharing and support the reproducibility and reuse of our findings. Thank you for your valuable guidance.

3.We note that the grant information you provided in the ‘Funding Information’ and ‘Financial Disclosure’ sections do not match.

When you resubmit, please ensure that you provide the correct grant numbers for the awards you received for your study in the ‘Funding Information’ section.

**Response:** Thank you for pointing out this issue. We sincerely apologize for the inconsistency between the funding information provided in the ‘Funding Information’ and ‘Financial Disclosure’ sections. We have carefully rechecked all grant numbers and related information, and have now ensured that they are fully consistent and correctly presented in the ‘Funding Information’ section. We appreciate your patience and guidance.

4.Thank you for stating the following financial disclosure:

“This work was supported by the National Natural Science Foundation of China ( No. 82305181, No. 82405252 and No. 82474601), Shenzhen Basic Research Special Natural Science Foundation Plan Project (No.JCYJ20250604150049018), the Guangdong Basic and Applied Basic Research Foundation (No. 2025A1515011830), the Scientific Research Project of Traditional Chinese Medicine Bureau of Guangdong Province (No. 20251426), Special Project for Clinical and Basic Sci&Tech Innovation of Guangdong Medical University, Shenzhen Key Laboratory (No. ZDSYS20210623092000002).”

Please state what role the funders took in the study. If the funders had no role, please state: “The funders had no role in study design, data collection and analysis, decision to publish, or preparation of the manuscript.”

If this statement is not correct you must amend it as needed.

Please include this amended Role of Funder statement in your cover letter; we will change the online submission form on your behalf.

**Response:** We thank the editor for this reminder and your helpful advice. We have now prepared an amended statement that declares all sources of funding and support received during this study, in accordance with the instructions provided. The amended Role of Funder statement is as follows:“The funders had no role in study design, data collection and analysis, decision to publish, or preparation of the manuscript.” The revised Funding Statement has been included in the cover letter, and we kindly ask that you update the online submission form on our behalf. We appreciate your assistance.

5.Thank you for uploading your study’s underlying data set. Unfortunately, the repository you have noted in your Data Availability statement does not qualify as an acceptable data repository according to PLOS’s standards.

At this time, please upload the minimal data set necessary to replicate your study’s findings to a stable, public repository (such as figshare or Dryad) and provide us with the relevant URLs, DOIs, or accession numbers that may be used to access these data.For a list of recommended repositories and additional information on PLOS standards for data deposition, please see https://journals.plos.org/plosone/s/recommended-repositories.

**Response:** We thank the Editor for bringing this issue to our attention and apologize for initially selecting a repository that did not meet PLOS ONE’s data-sharing requirements.

In response to this comment, we have carefully reviewed the PLOS ONE guidelines on data deposition and have deposited the minimal underlying dataset necessary to reproduce the findings of our study in a stable, publicly accessible repository that complies with PLOS standards.

Specifically, all relevant data supporting the conclusions of this study have been uploaded to Figshare and are publicly available at:https://doi.org/10.6084/m9.figshare.32415090

The repository contains the minimal dataset required to replicate the analyses and verify the results reported in the manuscript. We have also updated the Data Availability Statement accordingly in the revised manuscript.

We believe that these revisions fully satisfy PLOS ONE’s requirements regarding data availability and reproducibility. Thank you for your valuable guidance.

6.Please include a separate caption for each figure in your manuscript.

**Response:** Thank you for your reminder. We have confirmed that the manuscript includes captions, which appear on a separate page after the references section. All captions are complete and consistent with the image files. We hope this resolves the issue.

7.If the reviewer comments include a recommendation to cite specific previously published works, please review and evaluate these publications to determine whether they are relevant and should be cited. There is no requirement to cite these works unless the editor has indicated otherwise.

[Note: HTML markup is below. Please do not edit.]

**Response:**We thank the reviewer for recommending these valuable references. We have carefully reviewed the suggested publications and found them to be highly relevant to our study. Accordingly, we have cited them in the revised manuscript as follows:

[Feng D et al. (2025), DOI: 10.1136/egastro-2025-100189] : Identified FABP5 as a differentially expressed gene in distinct macrophage subpopulations (C1q+ MoMFs) following liver injury. This finding directly supports our investigation of FABP5 in the liver immune microenvironment. We have cited this work in the Introduction (to establish the rationale for studying FABP5 in HCC immunity) and in the Discussion (to contextualize our findings within the broader framework of liver macrophage heterogeneity).

[Wen Y et al. (2025), DOI: 10.1136/egastro-2025-100202] : Comprehensively reviewed single-cell transcriptomic advances in liver injury and regeneration. We have cited this work in the Introduction to highlight the complexity of the liver immune microenvironment and the value of transcriptomic approaches for dissecting cell-type-specific roles.

The reference list has been updated accordingly, and all changes are highlighted in the revised manuscript. We are grateful to the reviewer for these valuable recommendations.

8.Please upload a copy of Supporting Information Figure S1 Fig which you refer to in your text on page 39.

**Response:** We apologize for the omission. We have resubmitted the revised manuscript package through the system, including the newly added Figure S1 file. Please let us know if any further adjustments are required.

If you need additional time or have any questions, we are happy to provide further clarification. We appreciate your handling of our submission and look forward to your feedback.

**-Reviewer 1**

1. The term “shapes” in the title is too strong and should be toned down.

**Response:**We thank the reviewer for this constructive suggestion. We agree that the word “shapes” may imply a degree of functional dominance that is not fully supported by our current data. To more accurately reflect the role of FABP5 as a regulator — rather than the sole determinant — of the immunosuppressive microenvironment, we have toned down the title as recommended.

The title has now been revised to:“FABP5 Regulates an Immunosuppressive Microenvironment in Hepatocellular Carcinoma Through the NF-κB/PD-L1 Axis.”

We believe this revised title is more precise and appropriately cautious. We sincerely appreciate the reviewer’s careful reading and helpful suggestion.

2. Introduction is overly focused on liver cancer epidemiology and is not well connected to the main scientific question. It should be streamlined with greater emphasis on the current knowledge gaps regarding FABP5, metabolic reprogramming, the tumor immune microenvironment.

**Response:**We thank the reviewer for this constructive and insightful comment. We agree that the original introduction placed excessive emphasis on liver cancer epidemiology and MASLD background, which distracted from the core scientific focus on FABP5, metabolic reprogramming, and the tumor immune microenvironment (TIME).

Streamlined the background: We have made minor deletions to the content related to epidemiology and MASLD.

Strengthened the connection to FABP5, metabolic reprogramming, and TIME: We have added a new paragraph that synthesizes recent evidence demonstrating FABP5 as a key node linking metabolic reprogramming to immune regulation (page 3-4, lines 61-72).

Explicitly stated the current knowledge gaps: While the revised introduction identifies FABP5 as a promising therapeutic target, it also makes clear that the specific mechanisms by which FABP5 regulates the immunosuppressive TIME in HCC—particularly through the NF-κB/PD-L1 axis—remain to be fully elucidated. This directly sets up the rationale for our study.

We believe the revised introduction is now more focused, directly connected to our main scientific question, and appropriately emphasizes the knowledge gaps regarding FABP5, metabolic reprogramming, and the TIME. All changes are highlighted in the revised manuscript. We are grateful for this guidance, which has significantly improved the quality of our introduction.

3.The criteria for the pan-cancer and LIHC-specific analyses should be described more clearly, including inclusion/exclusion criteria, the source of normal controls, and whether batch correction was performed. The authors should also specify the R packages, parameters, and software versions used.

Response: Thank you for this valuable suggestion.

We have substantially revised the Data Acquisition and Preprocessing section to provide a clearer description of sample selection, normal control sources, batch-effect correction procedures, and software details. Specifically:

Inclusion and exclusion criteria were added. Only samples with available RNA-seq expression profiles and corresponding clinical information were retained for downstream analyses. Samples lacking either expression data or clinical annotations were excluded.

Normal control sources were clarified. For pan-cancer analyses, normal tissues were obtained from TCGA matched normal samples when available. For LIHC-specific analyses, adjacent non-tumor liver tissues from TCGA-LIHC were used as controls.

Batch-effect correction was explicitly described. GEO datasets (GSE121248 and GSE174570) were integrated using the ComBat algorithm implemented in the sva package (v3.44.0), with tumor/normal status preserved as a biological variable while removing technical batch effects.

Software and package information were added. All analyses were conducted using R software (version 4.0.3). Key packages used in this study are now specified in the Methods section, including sva (v3.44.0), limma, DESeq2, clusterProfiler, org.Hs.eg.db, Seurat, MAESTRO, immunedeconv, forestplot, and rms.

These revisions have been incorporated into the revised manuscript (page 5-6, lines 100-115 and corresponding subsections).

4. The scRNA-seq section should provide the name/accession number of the TISCH dataset used. Since the single-cell analysis indicates that FABP5 is mainly expressed in fibroblasts and Mono/Macro populations, it is unclear why the downstream validation was restricted to a single tumor cell line, Huh7. Additional co-culture experiments would strengthen the manuscript and better support the claims regarding the immunosuppressive microenvironment.

**Response:** We sincerely thank the reviewer for this insightful comment.

We agree that the source of the single-cell dataset should be explicitly described. Accordingly, we have revised the Methods section to provide the name and accession number of the TISCH dataset used for the scRNA-seq analysis. The corresponding information has now been added in the revised manuscript.

We acknowledge the reviewer’s concern regarding the apparent discrepancy between the scRNA-seq findings and the subsequent functional experiments. The primary objective of our in vitro experiments was to determine whether FABP5 possesses intrinsic tumor-promoting activity in hepatocellular carcinoma cells. Therefore, we selected Huh7 cells, which exhibited relatively high FABP5 expression, to evaluate the effects of FABP5 knockdown on proliferation, migration, colony formation, and EMT-related markers.

We agree that the single-cell data indicate that FABP5 is predominantly expressed in fibroblasts and monocyte/macrophage populations within the tumor microenvironment, suggesting that FABP5 may also participate in stromal-immune regulation beyond its direct effects on tumor cells.

To further address this important issue, we performed additional immune infiltration analyses using batch-corrected GEO hepatocellular carcinoma datasets. Specifically, the CIBERSORT algorithm was applied to estimate the abundance of 22 immune cell populations.

The newly added results demonstrated that FABP5 expression was significantly positively correlated with M0 macrophage infiltration (*p* = 0.004), while negatively correlated with M1 macrophage infiltration (*p* = 0.015) and naïve B-cell abundance (*p* = 0.009). These findings provide independent evidence supporting an association between elevated FABP5 expression and an immunosuppressive immune landscape. The related information has been added to the Results section, together with corresponding figures and methodological details (page 9-10, lines 194-198) (page 19-20, lines 407-422).

Following the reviewer’s suggestion, we have substantially expanded the Discussion section. We now emphasize that FABP5 is predominantly enriched in fibroblasts and monocyte/macrophage populations and may contribute to tumor progression through regulation of stromal and immune cell functions. The newly added CIBERSORT results further support a role for FABP5 in shaping an immunosuppressive tumor microenvironment by influencing macrophage polarization and adaptive immune-cell infiltration.

We fully agree that co-culture systems involving HCC cells and macrophages/fibroblasts would provide stronger mechanistic evidence. The experiments have been acknowledged as an important direction for future investigation. We have added this point to the revised Discussion and Limitations sections (page 33, lines 713-715, 718-720).

We appreciate the reviewer’s suggestion, which has significantly improved the rigor and interpretation of our study.

5. The Methods section should include details for the PCR/qPCR assay, wound-healing assay, FABP5 overexpression protocol, and Bay 11-7082 treatment conditions.

**Response:**We thank the reviewer for this careful reading. We agree that providing detailed experimental conditions is essential for reproducibility. Accordingly, we have substantially expanded the Materials and Methods section to include all requested details. The specific additions are summarized below and highlighted in the revised manuscript:

PCR/qPCR assay (page 12-13, Lines 260-275, subsection “RNA extraction and quantitative real-time PCR”)

Wound-healing assay (page 13, Lines 276-285, subsection “Wound-healing assay”)

FABP5 overexpression protocol (page 7-8, Lines 148-156, subsection “Cell culture and transfection”)

Bay 11-7082 treatment (page 14, Lines 286-299, subsection “Bay 11-7082 treatment”)

All the aforementioned revisions are included in the manuscript. We would like to express our sincere gratitude to the reviewers for their assistance in enhancing the completeness of the paper.

6. Please provide complete information for all antibodies used in the Western blot experiments.

**Response:**We thank the reviewer for this important reminder. To ensure reproducibility, we have now provided complete information for all antibodies used in the Western blot experiments in the revised Materials and Methods section.This information has been added to the “2.9 Western blot” section (page 8-9, Lines 175–192). All modifications are highlighted in the revised manuscript. We believe this level of detail now meets the methodological reporting standards of PLOS ONE. We appreciate the reviewers' attention to the completeness of the report.

7. The Figure 1 legend does not seem fully consistent with the main text. Please verify the panel labels and corresponding descriptions and check the consistency of the figure legends, panel labels, and text descriptions.

**Response:** We thank the reviewer for identifying this inconsistency. We have carefully cross-checked Figure 1, its legend, and all corresponding mentions in the main text (including the Results section and any figure citations elsewhere). Errors in the legends of Figures C and D of Figure 1 were identified and corrected. All corrections have been made in the revised manuscript, and the Figure 1 legend now accurately reflects the figure content and matches the descriptions in the main text. The changes are highlighted in the revised file. We apologize for any confusion these inconsistencies may have caused and thank the reviewer for the careful check.

8. There are two sections labeled 3.8, and section 3.9 is missing. there is no direct evidence of interaction, such as co-IP, pull-down, or colocalization assays. Therefore, describing these interactions as “forms stable complexes” is not appropriate.

**Response:** We thank the reviewer for this rigorous and constructive critique. We fully acknowledge two issues:

Section numbering error: The second “3.8” should have been “3.9”. We have corrected this in the revised manuscript. The section on CZS-241 is now properly labeled as “3.9”.

Overstatement of computational findings: We agree with the reviewer that our current data are solely computational predictions (molecular docking and MD simulations) and do not provide direct biochemical evidence of physical interaction between FABP5 and NF-κB components (e.g., IκBα, IKKβ, p65). Therefore, statements such as “forms stable complexes” and “supporting strong and specific interactions” are indeed inappropriate and misleading.

To address this concern, we have substantially revised Section 3.8 as follows: Toned down all claims from definitive and removed some similar overstatements. Added explicit limitations at the end of the section: “These computational predictions require experimental validation (e.g., co-immunoprecipitation，pull-down assays or confocal colocalization assays) in future studies.”(page 24, lines 507-512)

All changes are highlighted in the revised manuscript. We are grateful to the reviewer for preventing us from overinterpreting computational data. We believe these revisions now accurately represent the exploratory, hypothesis-generating nature of our computational analyses. We respectfully request the reviewer’s understanding that direct interaction assays are beyond the scope of the current revision, but we have clearly stated this as a critical direction for future research.

9.The Discussion states that “free energy further confirmed the presence of a predominant low-energy basin,” but the Results section does not clearly present a corresponding free-energy landscape analysis.

**Response:** We thank the reviewer for this important observation.

We agree that the original manuscript referred to a predominant low-energy basin in the Discussion without providing a corresponding free-energy landscape (FEL) analysis in the Results and Methods sections.

To address this issue, we have now performed and incorporated FEL analyses based on the molecular dynamics trajectories of the FABP5–CZS-241 complex from two independent simulation runs. The FELs were generated using the GROMACS gmx sham module, with RMSD (PC1) and radius of gyration (Rg, PC2) selected as reaction coordinates.

The corresponding FEL plots have been added to Fig 9N, with the left panel representing replicate 1 and the right panel representing replicate 2. Both simulations revealed a predominant low-energy basin centered around RMSD ≈ 0.18 nm and Rg ≈ 2.25–2.26 nm, indicating that the FABP5–CZS-241 complex converged toward a stable conformational state during the simulations (page 26, lines 557-563).

In addition, a detailed description of the FEL methodology has been added to the Molecular Dynamics Simulation subsection of the Methods section (page 12, lines 257-259). The statement in the Discussion regarding the presence of a predominant low-energy basin is now directly supported by the newly added FEL analyses.

These revisions improve the consistency between the Results, Methods, and Discussion sections.
10. The reference list is relatively limited and could be strengthened. The following references may be cited where appropriate: 10.1136/egastro-2025-100189; 10.1136/egastro-2025-100202

**Response:**We thank the reviewer for these constructive suggestions to strengthen our reference list. We have carefully reviewed the two recommended articles and find them both highly relevant to our study. Specifically:

[Feng D et al. (2025), DOI: 10.1136/egastro-2025-100189] : Identified FABP5 as a differentially expressed gene in distinct macrophage subpopulations (C1q+ MoMFs) following liver injury. This finding directly supports our investigation of FABP5 in the liver immune microenvironment. We have cited this work in the Introduction (to establish the rationale for studying FABP5 in HCC immunity) and in the Discussion (to contextualize our findings within the broader framework of liver macrophage heterogeneity).

[Wen Y et al. (2025), DOI: 10.1136/egastro-2025-100202] : Comprehensively reviewed single-cell transcriptomic advances in liver injury and regeneration. We have cited this work in the Introduction to highlight the complexity of the liver immune microenvironment and the value of transcriptomic approaches for dissecting cell-type-specific roles.

We believe the addition of these two references enhances the scholarly foundation of our manuscript. All changes are highlighted in the revised reference list. We are grateful to the reviewer for bringing these relevant publications to our attention.
11. The manuscript is recommended for English language editing to improve the accuracy of expression.

**Response:**We thank the reviewer for this suggestion. The manuscript has now been carefully reviewed and edited by a native English-speaking colleague with expertise in the field (or: using AI-assisted language tools followed by manual verification). The language accuracy and clarity have been substantially improved throughout the manuscript. All changes are highlighted in the revised version. We trust the manuscript now meets the required language standards.
