# Supplementary material for: FABP5 regulates an immunosuppressive microenvironment in hepatocellular carcinoma through the NF-κB/PD-L1 axis
Source: PLoS One. 2026 Sep 29;21(9):e0356359. doi: 10.1371/journal.pone.0356359 (PMC13623383; doi:10.1371/journal.pone.0356359)
Supplement: Supplementary file 4 [file pone.0356359.s005.docx]

**Responses to Reviewers’ comments**

**Dear editor and reviewers:**

We extend our sincere gratitude for your valuable insights and professional guidance once again. On behalf of all co-authors, we appreciate your significant time and effort contributed to this manuscript. We have carefully reviewed all your feedback and made corresponding revisions. Each of your suggestions will be addressed in detail below. We hope the revised version now meets all publication requirements. Thank you once more for your support.

**Title:** FABP5 Regulates an Immunosuppressive Microenvironment in Hepatocellular Carcinoma Through the NF-κB/PD-L1 Axis.(Manuscript ID: PONE-D-26-15285R1 )

[Original title: FABP5 Shapes an Immunosuppressive Microenvironment in Hepatocellular Carcinoma Through the NF-κB/PD-L1 Axis.]

**-Journal Requirements**

1. Please provide the original files of western blot for checking.

**Response:** Thank you for the editor's reminder. All original, uncropped full‑membrane scans or film negatives of the western blot bands presented in the manuscript, along with molecular weight markers and sample labels, have been provided. These raw images have been compiled into a single PDF file labeled “original image of WB” and submitted as supplementary material in the submission system. Each membrane image is clearly annotated with the corresponding antibody information in accordance with journal policies. The files are fully accessible for verification purposes.We would like to once again express our sincere gratitude to the Academic Editor for the valuable and insightful comments.

2. Please review your reference list to ensure that it is complete and correct. If you have cited papers that have been retracted, please include the rationale for doing so in the manuscript text, or remove these references and replace them with relevant current references. Any changes to the reference list should be mentioned in the rebuttal letter that accompanies your revised manuscript. If you need to cite a retracted article, indicate the article’s retracted status in the References list and also include a citation and full reference for the retraction notice.

**Response:** Thank you for the valuable feedback provided by the editor. We have carefully reviewed our reference list to ensure that it is complete and correct. All cited articles have been verified, and we confirm that none of the references have been retracted. Therefore, no retraction notices are applicable, and no changes have been made to the reference list. The reference list remains as originally submitted and complies with the journal's requirements.

3. If you would like to make changes to your financial disclosure, please include your updated statement in your cover letter.

**Response:** Thank you for the editor's reminder. We confirm that our financial disclosure statement remains unchanged from the original submission, and no updates are necessary. Should the journal require any additional information, we are happy to provide it promptly.

4. If applicable, we recommend that you deposit your laboratory protocols in protocols.io to enhance the reproducibility of your results. Protocols.io assigns your protocol its own identifier (DOI) so that it can be cited independently in the future. For instructions see: [https://journals.plos.org/plosone/s/submission-guidelines#loc-laboratory-protocols](https://track.editorialmanager.com/CL0/https://journals.plos.org/plosone/s/submission-guidelines%23loc-laboratory-protocols/1/010f019f216893ee-f7b37b6b-6802-4ee2-8555-84163dc79cb2-000000/iLi1n5PDbGojdLTncgBzNEIEQPszou3DkwgBipDm57s=258). Additionally, PLOS ONE offers an option for publishing peer-reviewed Lab Protocol articles, which describe protocols hosted on protocols.io. Read more information on sharing protocols at [https://plos.org/protocols?utm_medium=editorial-email&utm_source=authorletters&utm_campaign=protocols](https://track.editorialmanager.com/CL0/https://plos.org/protocols?utm_medium=editorial-email&utm_source=authorletters&utm_campaign=protocols/1/010f019f216893ee-f7b37b6b-6802-4ee2-8555-84163dc79cb2-000000/iivmIb_ZbFfJ0-Eqm_vpCBQUWjnYTJfxOHonaMvWUWg=258).

**Response:** Our sincere gratitude to the editor for their constructive suggestions. We also acknowledge the journal’s recommendation to deposit laboratory protocols to protocols.io for improving research reproducibility. We will consider depositing our experimental protocols on this platform after formal acceptance of this work.

5. As the corresponding author, your ORCID iD is verified in the submission system and will appear in the published article. PLOS supports the use of ORCID, and we encourage all coauthors to register for an ORCID iD and use it as well. Please encourage your coauthors to verify their ORCID iD within the submission system before final acceptance, as unverified ORCID iDs will not appear in the published article. Only the individual author can complete the verification step; PLOS staff cannot verify ORCID iDs on behalf of authors.

**Response:** We sincerely thank the Academic Editor for the reminder regarding the ORCID iD verification requirement. In response, we confirm that the corresponding author’s ORCID iD has already been verified in the submission system. In addition, we have contacted all coauthors and strongly encouraged them to verify their own ORCID iDs through the submission system before final acceptance, in accordance with the journal’s policy.

**-Reviewer 1**

1. If the authors have adequately addressed your comments raised in a previous round of review and you feel that this manuscript is now acceptable for publication, you may indicate that here to bypass the “Comments to the Author” section, enter your conflict of interest statement in the “Confidential to Editor” section, and submit your "Accept" recommendation.

Reviewer 1: All comments have been addressed

**Response:** We are greatly thankful that the reviewer confirms all previous review concerns have been fully resolved. We have carefully polished the whole manuscript for minor editorial-level issues as the reviewer suggested, to further improve the readability and presentation quality of this work.

2. Is the manuscript technically sound, and do the data support the conclusions?

The manuscript must describe a technically sound piece of scientific research with data that supports the conclusions. Experiments must have been conducted rigorously, with appropriate controls, replication, and sample sizes. The conclusions must be drawn appropriately based on the data presented.

Reviewer 1: Yes

**Response:** We appreciate this positive recognition. All experiments in this study were designed with proper negative/positive controls, independent biological replicates and sufficient sample sizes. All experimental procedures were rigorously implemented following standard protocols, and all conclusions drawn are strictly supported by our original experimental data.

3. Has the statistical analysis been performed appropriately and rigorously?

Reviewer 1: Yes

**Response:** We thank the reviewer for the affirmation. All statistical methods applied in our study were selected rationally according to data distribution and experimental design. Statistical calculations, significance analysis and data visualization were completed with standardized statistical software, and we have double-checked all statistical results to ensure rigor and correctness.

4. Have the authors made all data underlying the findings in their manuscript fully available?

The [PLOS Data policy](http://track.editorialmanager.com/CL0/http://www.plosone.org/static/policies.action%23sharing/1/010f019f216893ee-f7b37b6b-6802-4ee2-8555-84163dc79cb2-000000/RHuc5JqKQv5RE1y-kzeh5zHe-9WSc5RwqhPKDwogRsI=258) requires authors to make all data underlying the findings described in their manuscript fully available without restriction, with rare exception (please refer to the Data Availability Statement in the manuscript PDF file). The data should be provided as part of the manuscript or its supporting information, or deposited to a public repository. For example, in addition to summary statistics, the data points behind means, medians and variance measures should be available. If there are restrictions on publicly sharing data—e.g. participant privacy or use of data from a third party—those must be specified.

Reviewer 1: Yes

**Response:** We appreciate this confirmation. All raw datasets, quantitative statistical source data and unprocessed experimental source images supporting our conclusions are preserved completely. In compliance with PLOS ONE data policy, we will provide original full-length western blot raw files for journal inspection in this revision submission, and we are ready to share all underlying original data upon reasonable request.

5. Is the manuscript presented in an intelligible fashion and written in standard English?

PLOS ONE does not copyedit accepted manuscripts, so the language in submitted articles must be clear, correct, and unambiguous. Any typographical or grammatical errors should be corrected at revision, so please note any specific errors here.

Reviewer 1: Yes

**Response:** We are grateful for this comment. During revision, we have carefully proofread the full text sentence by sentence to eliminate typographical mistakes, grammatical errors and awkward expressions, optimizing sentence logic and academic wording to conform to standard formal English writing requirements for biomedical research articles.

6. Review Comments to the Author

Please use the space provided to explain your answers to the questions above. You may also include additional comments for the author, including concerns about dual publication, research ethics, or publication ethics. (Please upload your review as an attachment if it exceeds 20,000 characters)

Reviewer 1: The revised manuscript has adequately addressed the major concerns raised during review and has been substantially improved. The remaining issues are minor and mainly editorial. I therefore support publication of the manuscript, subject to routine editorial polishing.

**Response:** We would like to express our sincere gratitude to the reviewer for the thorough and constructive evaluation of our manuscript over the two rounds of review. The insightful comments and suggestions have been extremely valuable in strengthening our work and improving the clarity of our presentation.

We are very pleased that the reviewer found our revisions satisfactory and confirmed that all concerns have been adequately addressed. We greatly appreciate the time and effort that the reviewer has dedicated to reviewing our submission. In accordance with your comment that only minor editorial-level issues remain, we have conducted comprehensive editorial polishing across the entire manuscript:

We carefully checked the consistency of terminology, abbreviations, gene/protein nomenclature (FABP5, NF-κB, PD-L1) throughout the full text to unify writing specifications;

We adjusted sentence structure, polished redundant expressions, and refined paragraph transitions to improve overall manuscript fluency and readability;

We re-examined figure legends, table annotations and reference formatting to match PLOS ONE formatting standards;

We cross-checked punctuation, spelling and grammatical details to eliminate trivial writing flaws.

After the above editorial revisions, we confirm there exists no dual publication risk, and all research was performed under complete compliance with relevant publication ethics and research ethics regulations.

Once again, we thank the reviewer for the positive feedback and invaluable guidance throughout this process.

7. PLOS authors have the option to publish the peer review history of their article ([what does this mean?](https://track.editorialmanager.com/CL0/https://journals.plos.org/plosone/s/editorial-and-peer-review-process%23loc-peer-review-history/1/010f019f216893ee-f7b37b6b-6802-4ee2-8555-84163dc79cb2-000000/yiLpuKHhYMZKqvWD-1236h4jkEsxztXONUwfJiqcDY8=258)). If published, this will include your full peer review and any attached files.

If you choose “no”, your identity will remain anonymous but your review may still be made public.

Do you want your identity to be public for this peer review? For information about this choice, including consent withdrawal, please see our [Privacy Policy](https://track.editorialmanager.com/CL0/https://www.plos.org/privacy-policy/1/010f019f216893ee-f7b37b6b-6802-4ee2-8555-84163dc79cb2-000000/4pGSo-UcYG7eD_IfyTAhyM86po5b0VFGdE7Sb20DBuw=258).

Reviewer 1: No

**Response:** We respect the reviewer’s choice to remain anonymous in this peer review process, and we have no relevant inquiries regarding this setting.
