# Supplementary material for: FABP5 regulates an immunosuppressive microenvironment in hepatocellular carcinoma through the NF-κB/PD-L1 axis
Source: PLoS One. 2026 Sep 29;21(9):e0356359. doi: 10.1371/journal.pone.0356359 (PMC13623383; doi:10.1371/journal.pone.0356359)
Supplement: Supplementary file 5 [file pone.0356359.s006.docx]

**Responses to Reviewers’ comments**

**Dear editor and reviewers:**

We extend our sincere gratitude for your valuable insights and professional guidance once again. On behalf of all co-authors, we appreciate your significant time and effort contributed to this manuscript. We have carefully reviewed all your feedback and made corresponding revisions. Each of your suggestions will be addressed in detail below. We hope the revised version now meets all publication requirements. Thank you once more for your support.

**Title:** FABP5 Regulates an Immunosuppressive Microenvironment in Hepatocellular Carcinoma Through the NF-κB/PD-L1 Axis.(Manuscript ID: PONE-D-26-15285R2 )

[Original title: FABP5 Shapes an Immunosuppressive Microenvironment in Hepatocellular Carcinoma Through the NF-κB/PD-L1 Axis.]

**-Journal Requirements**

1. If you would like to make changes to your financial disclosure, please include your updated statement in your cover letter. Guidelines for resubmitting your figure files are available below the reviewer comments at the end of this letter.

**Response:** We are grateful for the opportunity to revise our work. We have reviewed our financial disclosure statement and confirm that no changes are needed. The original statement remains accurate. We hope that the revised manuscript now meets the journal's requirements. Please do not hesitate to let us know if any further revisions are needed.

2.If the reviewer comments include a recommendation to cite specific previously published works, please review and evaluate these publications to determine whether they are relevant and should be cited. There is no requirement to cite these works unless the editor has indicated otherwise.

**Response:** Thank you for the valuable feedback provided by the editor. The reviewers did not provide any specific recommendations for additional citations. We have therefore maintained our reference list as originally submitted, with updates made only where necessary to address the scientific content of the reviews.

3.Please review your reference list to ensure that it is complete and correct. If you have cited papers that have been retracted, please include the rationale for doing so in the manuscript text, or remove these references and replace them with relevant current references. Any changes to the reference list should be mentioned in the rebuttal letter that accompanies your revised manuscript. If you need to cite a retracted article, indicate the article’s retracted status in the References list and also include a citation and full reference for the retraction notice.

**Response:** Thank you for the editor’s reminder. We have carefully reviewed our entire reference list. We confirm that none of the cited papers have been retracted. All references are complete, accurate, and relevant to the content of our manuscript.

4. Please provide the original, uncropped and minimally adjusted images supporting all blot and gel results reported in an article’s figures and supporting information files (see for more information: [https://journals.plos.org/plosone/s/figures#loc-blot-and-gel-reporting-requirements](https://journals.plos.org/plosone/s/figures#loc-blot-and-gel-reporting-requirements)).

**Response:** Our sincere gratitude to the editor for their constructive suggestions. We have compiled all original, uncropped, and minimally adjusted images supporting the blot and gel results presented in our manuscript’s figures and supporting information. These have been assembled into a single PDF file named S1 raw_images. This file has been uploaded as a Supporting Information file as per the journal’s guidelines. We confirm that all images comply with PLOS ONE’s blot and gel reporting requirements.

**-Reviewer**

**Response to reviewers:** We would like to express our sincere gratitude to the editor and the reviewers for taking the time to evaluate our manuscript. We greatly appreciate the thoughtful assessment of our work.The reviewers did not raise any specific concerns or requests for revision regarding the scientific content of our manuscript. We are very encouraged by this positive feedback.We trust that the revised version now meets the journal's standards for publication. Should the reviewers or editors have any further suggestions, we would be happy to address them promptly.
