# Supplementary material for: FABP5 regulates an immunosuppressive microenvironment in hepatocellular carcinoma through the NF-κB/PD-L1 axis
Source: PLoS One. 2026 Sep 29;21(9):e0356359. doi: 10.1371/journal.pone.0356359 (PMC13623383; doi:10.1371/journal.pone.0356359)
Supplement: Peer review history

# Peer review: Decision Letter 0

**DOI:** 10.1371/journal.pone.0356359.r001

**Authors:** Lanlan Chen

27 Apr 2026

PONE-D-26-15285 FABP5 shapes an immunosuppressive microenvironment in hepatocellular carcinoma through the NF-κB/PD-L1 axis PLOS One

Dear Dr. An,

Thank you for submitting your manuscript to PLOS ONE. After careful consideration, we feel that it has merit but does not fully meet PLOS ONE’s publication criteria as it currently stands. Therefore, we invite you to submit a revised version of the manuscript that addresses the points raised during the review process.

Please submit your revised manuscript by Jun 11 2026 11:59PM. If you will need more time than this to complete your revisions, please reply to this message or contact the journal office at plosone@plos.org. When you're ready to submit your revision, log on to https://www.editorialmanager.com/pone/ and select the 'Submissions Needing Revision' folder to locate your manuscript file.

Please include the following items when submitting your revised manuscript:

- A letter that responds to each point raised by the academic editor and reviewer(s). You should upload this letter as a separate file labeled 'Response to Reviewers'.
- A marked-up copy of your manuscript that highlights changes made to the original version. You should upload this as a separate file labeled 'Revised Manuscript with Track Changes'.
- An unmarked version of your revised paper without tracked changes. You should upload this as a separate file labeled 'Manuscript'.

If you would like to make changes to your financial disclosure, please include your updated statement in your cover letter. Guidelines for resubmitting your figure files are available below the reviewer comments at the end of this letter.

If applicable, we recommend that you deposit your laboratory protocols in protocols.io to enhance the reproducibility of your results. Protocols.io assigns your protocol its own identifier (DOI) so that it can be cited independently in the future. For instructions see: https://journals.plos.org/plosone/s/submission-guidelines#loc-laboratory-protocols. Additionally, PLOS ONE offers an option for publishing peer-reviewed Lab Protocol articles, which describe protocols hosted on protocols.io. Read more information on sharing protocols at https://plos.org/protocols?utm_medium=editorial-email&utm_source=authorletters&utm_campaign=protocols.

As the corresponding author, your ORCID iD is verified in the submission system and will appear in the published article. PLOS supports the use of ORCID, and we encourage all coauthors to register for an ORCID iD and use it as well. Please encourage your coauthors to verify their ORCID iD within the submission system before final acceptance, as unverified ORCID iDs will not appear in the published article. *Only* the individual author can complete the verification step; PLOS staff *cannot* verify ORCID iDs on behalf of authors.

We look forward to receiving your revised manuscript.

Kind regards,

Lanlan Chen

Academic Editor

PLOS One

Journal Requirements:

When submitting your revision, we need you to address these additional requirements.

1. Please ensure that your manuscript meets PLOS ONE's style requirements, including those for file naming. The PLOS ONE style templates can be found at

https://journals.plos.org/plosone/s/file?id=wjVg/PLOSOne_formatting_sample_main_body.pdf and

https://journals.plos.org/plosone/s/file?id=ba62/PLOSOne_formatting_sample_title_authors_affiliations.pdf

2. Please note that PLOS One has specific guidelines on code sharing for submissions in which author-generated code underpins the findings in the manuscript. In these cases, we expect all author-generated code to be made available without restrictions upon publication of the work. Please review our guidelines at https://journals.plos.org/plosone/s/materials-and-software-sharing#loc-sharing-code and ensure that your code is shared in a way that follows best practice and facilitates reproducibility and reuse.

3. We note that the grant information you provided in the ‘Funding Information’ and ‘Financial Disclosure’ sections do not match.

When you resubmit, please ensure that you provide the correct grant numbers for the awards you received for your study in the ‘Funding Information’ section.

4. Thank you for stating the following financial disclosure:

“This work was supported by the National Natural Science Foundation of China ( No. 82305181, No. 82405252 and No. 82474601), Shenzhen Basic Research Special Natural Science Foundation Plan Project (No.JCYJ20250604150049018), the Guangdong Basic and Applied Basic Research Foundation (No. 2025A1515011830), the Scientific Research Project of Traditional Chinese Medicine Bureau of Guangdong Province (No. 20251426), Special Project for Clinical and Basic Sci&Tech Innovation of Guangdong Medical University, Shenzhen Key Laboratory (No. ZDSYS20210623092000002).”

Please state what role the funders took in the study.  If the funders had no role, please state: "The funders had no role in study design, data collection and analysis, decision to publish, or preparation of the manuscript."

If this statement is not correct you must amend it as needed.

Please include this amended Role of Funder statement in your cover letter; we will change the online submission form on your behalf.

5. Thank you for uploading your study's underlying data set. Unfortunately, the repository you have noted in your Data Availability statement does not qualify as an acceptable data repository according to PLOS's standards.

At this time, please upload the minimal data set necessary to replicate your study's findings to a stable, public repository (such as figshare or Dryad) and provide us with the relevant URLs, DOIs, or accession numbers that may be used to access these data. For a list of recommended repositories and additional information on PLOS standards for data deposition, please see https://journals.plos.org/plosone/s/recommended-repositories.

6. Please include a separate caption for each figure in your manuscript.

7. If the reviewer comments include a recommendation to cite specific previously published works, please review and evaluate these publications to determine whether they are relevant and should be cited. There is no requirement to cite these works unless the editor has indicated otherwise.

[Note: HTML markup is below. Please do not edit.]

Reviewers' comments:

Reviewer's Responses to Questions

**Comments to the Author**

1. Is the manuscript technically sound, and do the data support the conclusions?

The manuscript must describe a technically sound piece of scientific research with data that supports the conclusions. Experiments must have been conducted rigorously, with appropriate controls, replication, and sample sizes. The conclusions must be drawn appropriately based on the data presented.

Reviewer #1: Partly

**********

2. Has the statistical analysis been performed appropriately and rigorously?

Reviewer #1: Yes

**********

3. Have the authors made all data underlying the findings in their manuscript fully available?

The PLOS Data policy requires authors to make all data underlying the findings described in their manuscript fully available without restriction, with rare exception (please refer to the Data Availability Statement in the manuscript PDF file). The data should be provided as part of the manuscript or its supporting information, or deposited to a public repository. For example, in addition to summary statistics, the data points behind means, medians and variance measures should be available. If there are restrictions on publicly sharing data—e.g. participant privacy or use of data from a third party—those must be specified.

Reviewer #1: Yes

**********

4. Is the manuscript presented in an intelligible fashion and written in standard English?

PLOS ONE does not copyedit accepted manuscripts, so the language in submitted articles must be clear, correct, and unambiguous. Any typographical or grammatical errors should be corrected at revision, so please note any specific errors here.

Reviewer #1: No

**********

5. Review Comments to the Author

Please use the space provided to explain your answers to the questions above. You may also include additional comments for the author, including concerns about dual publication, research ethics, or publication ethics. (Please upload your review as an attachment if it exceeds 20,000 characters)

Reviewer #1: 1. The term “shapes” in the title is too strong and should be toned down.

2. Introduction is overly focused on liver cancer epidemiology and is not well connected to the main scientific question. It should be streamlined with greater emphasis on the current knowledge gaps regarding FABP5, metabolic reprogramming, the tumor immune microenvironment.

3. The criteria for the pan-cancer and LIHC-specific analyses should be described more clearly, including inclusion/exclusion criteria, the source of normal controls, and whether batch correction was performed. The authors should also specify the R packages, parameters, and software versions used.

4. The scRNA-seq section should provide the name/accession number of the TISCH dataset used. Since the single-cell analysis indicates that FABP5 is mainly expressed in fibroblasts and Mono/Macro populations, it is unclear why the downstream validation was restricted to a single tumor cell line, Huh7. Additional co-culture experiments would strengthen the manuscript and better support the claims regarding the immunosuppressive microenvironment.

5. The Methods section should include details for the PCR/qPCR assay, wound-healing assay, FABP5 overexpression protocol, and Bay 11-7082 treatment conditions.

6. Please provide complete information for all antibodies used in the Western blot experiments.

7. The Figure 1 legend does not seem fully consistent with the main text. Please verify the panel labels and corresponding descriptions and check the consistency of the figure legends, panel labels, and text descriptions.

8. There are two sections labeled 3.8, and section 3.9 is missing. there is no direct evidence of interaction, such as co-IP, pull-down, or colocalization assays. Therefore, describing these interactions as “forms stable complexes” is not appropriate.

9. The Discussion states that “free energy further confirmed the presence of a predominant low-energy basin,” but the Results section does not clearly present a corresponding free-energy landscape analysis.

10. The reference list is relatively limited and could be strengthened. The following references may be cited where appropriate: 10.1136/egastro-2025-100189; 10.1136/egastro-2025-100202

11. The manuscript is recommended for English language editing to improve the accuracy of expression.

**********

6. PLOS authors have the option to publish the peer review history of their article (what does this mean?). If published, this will include your full peer review and any attached files.

If you choose “no”, your identity will remain anonymous but your review may still be made public.

**Do you want your identity to be public for this peer review?** For information about this choice, including consent withdrawal, please see our Privacy Policy.

Reviewer #1: No

**********

[NOTE: If reviewer comments were submitted as an attachment file, they will be attached to this email and accessible via the submission site. Please log into your account, locate the manuscript record, and check for the action link "View Attachments". If this link does not appear, there are no attachment files.]

To ensure your figures meet our technical requirements, please review our figure guidelines: https://journals.plos.org/plosone/s/figures

You may also use PLOS’s free figure tool, NAAS, to help you prepare publication quality figures: https://journals.plos.org/plosone/s/figures#loc-tools-for-figure-preparation.

NAAS will assess whether your figures meet our technical requirements by comparing each figure against our figure specifications.

# Author comment: Author response to Decision Letter 1

**DOI:** 10.1371/journal.pone.0356359.r002

24 Jun 2026

Responses to Reviewers’ comments

Dear editor and reviewers:

We sincerely thank you for your thoughtful comments and professional guidance. On behalf of all co-authors, I greatly appreciate the time and effort you have dedicated to our manuscript. We have carefully considered all the points raised and have revised the manuscript accordingly. Below, we provide a point-by-point response to each comment. We hope that the revised version is now suitable for publication. Thank you very much.

Title:FABP5 Regulates an Immunosuppressive Microenvironment in Hepatocellular Carcinoma Through the NF-κB/PD-L1 Axis.(Manuscript ID: PONE-D-26-15285 )

[Original title:FABP5 Shapes an Immunosuppressive Microenvironment in Hepatocellular Carcinoma Through the NF-κB/PD-L1 Axis.]

-Journal Requirements

1.Please ensure that your manuscript meets PLOS ONE’s style requirements, including those for file naming. The PLOS ONE style templates can be found at

https://journals.plos.org/plosone/s/file?id=wjVg/PLOSOne_formatting_sample_main_body.pdf and

https://journals.plos.org/plosone/s/file?id=ba62/PLOSOne_formatting_sample_title_authors_affiliations.pdf.

Response: We apologize for not adhering strictly to PLOS ONE’s style requirements in the initial submission. We have now carefully followed the guidelines and the provided templates (including the main body template and the title/authors/affiliations template) to reformat the manuscript accordingly. Specifically, we have adjusted the formatting of the title page, headings, line spacing, font, and reference style to comply with the journal’s standards. In addition, we have renamed all supplementary files according to PLOS ONE’s file naming conventions. A revised version of the manuscript with all these formatting corrections has been uploaded. We sincerely hope that the revised manuscript now meets the journal’s requirements.

2.Please note that PLOS One has specific guidelines on code sharing for submissions in which author-generated code underpins the findings in the manuscript. In these cases, we expect all author-generated code to be made available without restrictions upon publication of the work. Please review our guidelines at https://journals.plos.org/plosone/s/materials-and-software-sharing#loc-sharing-code and ensure that your code is shared in a way that follows best practice and facilitates reproducibility and reuse.

Response: We thank the Editor for highlighting the journal’s code-sharing requirements and fully recognize the importance of making computational methods openly available to ensure reproducibility and transparency.

To comply with the PLOS ONE Materials and Software Sharing Policy, we have made all author-generated code and relevant analysis outputs publicly available.

Specifically:

• The datasets used in this study were obtained from publicly accessible repositories, including The Cancer Genome Atlas (TCGA; https://portal.gdc.cancer.gov) and the Cancer Cell Line Encyclopedia (CCLE; https://depmap.org/portal/).

• The R script developed and used in this study has been deposited in the Figshare repository (https://doi.org/10.6084/m9.figshare.32415090) under the filename “FABP5_Code.R” and is freely accessible without restriction.

• The bioinformatics analysis results generated during the study have been deposited in the Figshare repository under the filename “Data for Bioinformatic Analysis.zip”.

• Molecular dynamics simulation data have also been deposited in the Figshare repository (DOI: https://doi.org/10.6084/m9.figshare.32415090) under the collection “Molecular Dynamics Simulations”.

The revised manuscript has been updated accordingly in the Data Availability Statement. We believe that the deposited code, data, and simulation files satisfy the PLOS ONE requirements for code sharing and support the reproducibility and reuse of our findings. Thank you for your valuable guidance.

3.We note that the grant information you provided in the ‘Funding Information’ and ‘Financial Disclosure’ sections do not match.

When you resubmit, please ensure that you provide the correct grant numbers for the awards you received for your study in the ‘Funding Information’ section.

Response: Thank you for pointing out this issue. We sincerely apologize for the inconsistency between the funding information provided in the ‘Funding Information’ and ‘Financial Disclosure’ sections. We have carefully rechecked all grant numbers and related information, and have now ensured that they are fully consistent and correctly presented in the ‘Funding Information’ section. We appreciate your patience and guidance.

4.Thank you for stating the following financial disclosure:

“This work was supported by the National Natural Science Foundation of China ( No. 82305181, No. 82405252 and No. 82474601), Shenzhen Basic Research Special Natural Science Foundation Plan Project (No.JCYJ20250604150049018), the Guangdong Basic and Applied Basic Research Foundation (No. 2025A1515011830), the Scientific Research Project of Traditional Chinese Medicine Bureau of Guangdong Province (No. 20251426), Special Project for Clinical and Basic Sci&Tech Innovation of Guangdong Medical University, Shenzhen Key Laboratory (No. ZDSYS20210623092000002).”

Please state what role the funders took in the study. If the funders had no role, please state: “The funders had no role in study design, data collection and analysis, decision to publish, or preparation of the manuscript.”

If this statement is not correct you must amend it as needed.

Please include this amended Role of Funder statement in your cover letter; we will change the online submission form on your behalf.

Response: We thank the editor for this reminder and your helpful advice. We have now prepared an amended statement that declares all sources of funding and support received during this study, in accordance with the instructions provided. The amended Role of Funder statement is as follows:“The funders had no role in study design, data collection and analysis, decision to publish, or preparation of the manuscript.” The revised Funding Statement has been included in the cover letter, and we kindly ask that you update the online submission form on our behalf. We appreciate your assistance.

5.Thank you for uploading your study’s underlying data set. Unfortunately, the repository you have noted in your Data Availability statement does not qualify as an acceptable data repository according to PLOS’s standards.

At this time, please upload the minimal data set necessary to replicate your study’s findings to a stable, public repository (such as figshare or Dryad) and provide us with the relevant URLs, DOIs, or accession numbers that may be used to access these data.For a list of recommended repositories and additional information on PLOS standards for data deposition, please see https://journals.plos.org/plosone/s/recommended-repositories.

Response: We thank the Editor for bringing this issue to our attention and apologize for initially selecting a repository that did not meet PLOS ONE’s data-sharing requirements.

In response to this comment, we have carefully reviewed the PLOS ONE guidelines on data deposition and have deposited the minimal underlying dataset necessary to reproduce the findings of our study in a stable, publicly accessible repository that complies with PLOS standards.

Specifically, all relevant data supporting the conclusions of this study have been uploaded to Figshare and are publicly available at: https://doi.org/10.6084/m9.figshare.32415090

The repository contains the minimal dataset required to replicate the analyses and verify the results reported in the manuscript. We have also updated the Data Availability Statement accordingly in the revised manuscript.

We believe that these revisions fully satisfy PLOS ONE’s requirements regarding data availability and reproducibility. Thank you for your valuable guidance.

6.Please include a separate caption for each figure in your manuscript.

Response: Thank you for your reminder. We have confirmed that the manuscript includes captions, which appear on a separate page after the references section. All captions are complete and consistent with the image files. We hope this resolves the issue.

7.If the reviewer comments include a recommendation to cite specific previously published works, please review and evaluate these publications to determine whether they are relevant and should be cited. There is no requirement to cite these works unless the editor has indicated otherwise.

[Note: HTML markup is below. Please do not edit.]

Response:We thank the reviewer for recommending these valuable references. We have carefully reviewed the suggested publications and found them to be highly relevant to our study. Accordingly, we have cited them in the revised manuscript as follows:

[Feng D et al. (2025), DOI: 10.1136/egastro-2025-100189] : Identified FABP5 as a differentially expressed gene in distinct macrophage subpopulations (C1q+ MoMFs) following liver injury. This finding directly supports our investigation of FABP5 in the liver immune microenvironment. We have cited this work in the Introduction (to establish the rationale for studying FABP5 in HCC immunity) and in the Discussion (to contextualize our findings within the broader framework of liver macrophage heterogeneity).

[Wen Y et al. (2025), DOI: 10.1136/egastro-2025-100202] : Comprehensively reviewed single-cell transcriptomic advances in liver injury and regeneration. We have cited this work in the Introduction to highlight the complexity of the liver immune microenvironment and the value of transcriptomic approaches for dissecting cell-type-specific roles.

The reference list has been updated accordingly, and all changes are highlighted in the revised manuscript. We are grateful to the reviewer for these valuable recommendations.

-Reviewer 1

1. The term “shapes” in the title is too strong and should be toned down.

Response:We thank the reviewer for this constructive suggestion. We agree that the word “shapes” may imply a degree of functional dominance that is not fully supported by our current data. To more accurately reflect the role of FABP5 as a regulator — rather than the sole determinant — of the immunosuppressive microenvironment, we have toned down the title as recommended.

The title has now been revised to:“FABP5 Regulates an Immunosuppressive Microenvironment in Hepatocellular Carcinoma Through the NF-κB/PD-L1 Axis.”

We believe this revised title is more precise and appropriately cautious. We sincerely appreciate the reviewer’s careful reading and helpful suggestion.

2. Introduction is overly focused on liver cancer epidemiology and is not well connected to the main scientific question. It should be streamlined with greater emphasis on the current knowledge gaps regarding FABP5, metabolic reprogramming, the tumor immune microenvironment.

Response:We thank the reviewer for this constructive and insightful comment. We agree that the original introduction placed excessive emphasis on liver cancer epidemiology and MASLD background, which distracted from the core scientific focus on FABP5, metabolic reprogramming, and the tumor immune microenvironment (TIME).

Streamlined the background: We have made minor deletions to the content related to epidemiology and MASLD.

Strengthened the connection to FABP5, metabolic reprogramming, and TIME: We have added a new paragraph that synthesizes recent evidence demonstrating FABP5 as a key node linking metabolic reprogramming to immune regulation (page 3-4, lines 61-72).

Explicitly stated the current knowledge gaps: While the revised introduction identifies FABP5 as a promising therapeutic target, it also makes clear that the specific mechanisms by which FABP5 regulates the immunosuppressive TIME in HCC—particularly through the NF-κB/PD-L1 axis—remain to be fully elucidated. This directly sets up the rationale for our study.

We believe the revised introduction is now more focused, directly connected to our main scientific question, and appropriately emphasizes the knowledge gaps regarding FABP5, metabolic reprogramming, and the TIME. All changes are highlighted in the revised manuscript. We are grateful for this guidance, which has significantly improved the quality of our introduction.

3.The criteria for the pan-cancer and LIHC-specific analyses should be described more clearly, including inclusion/exclusion criteria, the source of normal controls, and whether batch correction was performed. The authors should also specify the R packages, parameters, and software versions used.

Response: Thank you for this valuable suggestion.

We have substantially revised the Data Acquisition and Preprocessing section to provide a clearer description of sample selection, normal control sources, batch-effect correction procedures, and software details. Specifically:

Inclusion and exclusion criteria were added. Only samples with available RNA-seq expression profiles and corresponding clinical information were retained for downstream analyses. Samples lacking either expression data or clinical annotations were excluded.

Normal control sources were clarified. For pan-cancer analyses, normal tissues were obtained from TCGA matched normal samples when available. For LIHC-specific analyses, adjacent non-tumor liver tissues from TCGA-LIHC were used as controls.

Batch-effect correction was explicitly described. GEO datasets (GSE121248 and GSE174570) were integrated using the ComBat algorithm implemented in the sva package (v3.44.0), with tumor/normal status preserved as a biological variable while removing technical batch effects.

Software and package information were added. All analyses were conducted using R software (version 4.0.3). Key packages used in this study are now specified in the Methods section, including sva (v3.44.0), limma, DESeq2, clusterProfiler, org.Hs.eg.db, Seurat, MAESTRO, immunedeconv, forestplot, and rms.

These revisions have been incorporated into the revised manuscript (page 5-6, lines 100-115 and corresponding subsections).

4. The scRNA-seq section should provide the name/accession number of the TISCH dataset used. Since the single-cell analysis indicates that FABP5 is mainly expressed in fibroblasts and Mono/Macro populations, it is unclear why the downstream validation was restricted to a single tumor cell line, Huh7. Additional co-culture experiments would strengthen the manuscript and better support the claims regarding the immunosuppressive microenvironment.

Response: We sincerely thank the reviewer for this insightful comment.

We agree that the source of the single-cell dataset should be explicitly described. Accordingly, we have revised the Methods section to provide the name and accession number of the TISCH dataset used for the scRNA-seq analysis. The corresponding information has now been added in the revised manuscript.

We acknowledge the reviewer’s concern regarding the apparent discrepancy between the scRNA-seq findings and the subsequent functional experiments. The primary objective of our in vitro experiments was to determine whether FABP5 possesses intrinsic tumor-promoting activity in hepatocellular carcinoma cells. Therefore, we selected Huh7 cells, which exhibited relatively high FABP5 expression, to evaluate the effects of FABP5 knockdown on proliferation, migration, colony formation, and EMT-related markers.

We agree that the single-cell data indicate that FABP5 is predominantly expressed in fibroblasts and monocyte/macrophage populations within the tumor microenvironment, suggesting that FABP5 may also participate in stromal-immune regulation beyond its direct effects on tumor cells.

To further address this important issue, we performed additional immune infiltration analyses using batch-corrected GEO hepatocellular carcinoma datasets. Specifically, the CIBERSORT algorithm was applied to estimate the abundance of 22 immune cell populations.

The newly added results demonstrated that FABP5 expression was significantly positively correlated with M0 macrophage infiltration (p = 0.004), while negatively correlated with M1 macrophage infiltration (p = 0.015) and naïve B-cell abundance (p = 0.009). These findings provide independent evidence supporting an association between elevated FABP5 express

Attachment Submitted filename: Response to reviewers.docx

# Peer review: Decision Letter 1

**DOI:** 10.1371/journal.pone.0356359.r003

**Authors:** Lanlan Chen

2 Jul 2026

PONE-D-26-15285R1

FABP5 Regulates an Immunosuppressive Microenvironment in Hepatocellular Carcinoma Through the NF-κB/PD-L1 Axis

PLOS One

Dear Dr. An,

Thank you for submitting your manuscript to PLOS ONE. After careful consideration, we feel that it has merit but does not fully meet PLOS ONE’s publication criteria as it currently stands. Therefore, we invite you to submit a revised version of the manuscript that addresses the points raised during the review process.

Please provide the original files of western blot for checking.

Please submit your revised manuscript by Aug 15 2026 11:59PM. If you will need more time than this to complete your revisions, please reply to this message or contact the journal office at plosone@plos.org. When you're ready to submit your revision, log on to https://www.editorialmanager.com/pone/ and select the 'Submissions Needing Revision' folder to locate your manuscript file.

Please include the following items when submitting your revised manuscript:

- A letter that responds to each point raised by the academic editor and reviewer(s). You should upload this letter as a separate file labeled 'Response to Reviewers'.
- A marked-up copy of your manuscript that highlights changes made to the original version. You should upload this as a separate file labeled 'Revised Manuscript with Track Changes'.
- An unmarked version of your revised paper without tracked changes. You should upload this as a separate file labeled 'Manuscript'.

If you would like to make changes to your financial disclosure, please include your updated statement in your cover letter. Guidelines for resubmitting your figure files are available below the reviewer comments at the end of this letter.

If applicable, we recommend that you deposit your laboratory protocols in protocols.io to enhance the reproducibility of your results. Protocols.io assigns your protocol its own identifier (DOI) so that it can be cited independently in the future. For instructions see: https://journals.plos.org/plosone/s/submission-guidelines#loc-laboratory-protocols. Additionally, PLOS ONE offers an option for publishing peer-reviewed Lab Protocol articles, which describe protocols hosted on protocols.io. Read more information on sharing protocols at https://plos.org/protocols?utm_medium=editorial-email&utm_source=authorletters&utm_campaign=protocols.

As the corresponding author, your ORCID iD is verified in the submission system and will appear in the published article. PLOS supports the use of ORCID, and we encourage all coauthors to register for an ORCID iD and use it as well. Please encourage your coauthors to verify their ORCID iD within the submission system before final acceptance, as unverified ORCID iDs will not appear in the published article. *Only* the individual author can complete the verification step; PLOS staff *cannot* verify ORCID iDs on behalf of authors.

We look forward to receiving your revised manuscript.

Kind regards,

Lanlan Chen

Academic Editor

PLOS One

Journal Requirements:

If the reviewer comments include a recommendation to cite specific previously published works, please review and evaluate these publications to determine whether they are relevant and should be cited. There is no requirement to cite these works unless the editor has indicated otherwise.

Please review your reference list to ensure that it is complete and correct. If you have cited papers that have been retracted, please include the rationale for doing so in the manuscript text, or remove these references and replace them with relevant current references. Any changes to the reference list should be mentioned in the rebuttal letter that accompanies your revised manuscript. If you need to cite a retracted article, indicate the article’s retracted status in the References list and also include a citation and full reference for the retraction notice.

[Note: HTML markup is below. Please do not edit.]

Reviewers' comments:

Reviewer's Responses to Questions

**Comments to the Author**

1. If the authors have adequately addressed your comments raised in a previous round of review and you feel that this manuscript is now acceptable for publication, you may indicate that here to bypass the “Comments to the Author” section, enter your conflict of interest statement in the “Confidential to Editor” section, and submit your "Accept" recommendation.

Reviewer #1: All comments have been addressed

**********

2. Is the manuscript technically sound, and do the data support the conclusions?

The manuscript must describe a technically sound piece of scientific research with data that supports the conclusions. Experiments must have been conducted rigorously, with appropriate controls, replication, and sample sizes. The conclusions must be drawn appropriately based on the data presented.

Reviewer #1: Yes

**********

3. Has the statistical analysis been performed appropriately and rigorously?

Reviewer #1: Yes

**********

4. Have the authors made all data underlying the findings in their manuscript fully available?

The PLOS Data policy requires authors to make all data underlying the findings described in their manuscript fully available without restriction, with rare exception (please refer to the Data Availability Statement in the manuscript PDF file). The data should be provided as part of the manuscript or its supporting information, or deposited to a public repository. For example, in addition to summary statistics, the data points behind means, medians and variance measures should be available. If there are restrictions on publicly sharing data—e.g. participant privacy or use of data from a third party—those must be specified.

Reviewer #1: Yes

**********

5. Is the manuscript presented in an intelligible fashion and written in standard English?

PLOS ONE does not copyedit accepted manuscripts, so the language in submitted articles must be clear, correct, and unambiguous. Any typographical or grammatical errors should be corrected at revision, so please note any specific errors here.

Reviewer #1: Yes

**********

6. Review Comments to the Author

Please use the space provided to explain your answers to the questions above. You may also include additional comments for the author, including concerns about dual publication, research ethics, or publication ethics. (Please upload your review as an attachment if it exceeds 20,000 characters)

Reviewer #1: The revised manuscript has adequately addressed the major concerns raised during review and has been substantially improved. The remaining issues are minor and mainly editorial. I therefore support publication of the manuscript, subject to routine editorial polishing.

**********

7. PLOS authors have the option to publish the peer review history of their article (what does this mean?). If published, this will include your full peer review and any attached files.

If you choose “no”, your identity will remain anonymous but your review may still be made public.

**Do you want your identity to be public for this peer review?** For information about this choice, including consent withdrawal, please see our Privacy Policy.

Reviewer #1: No

**********

[NOTE: If reviewer comments were submitted as an attachment file, they will be attached to this email and accessible via the submission site. Please log into your account, locate the manuscript record, and check for the action link "View Attachments". If this link does not appear, there are no attachment files.]

To ensure your figures meet our technical requirements, please review our figure guidelines: https://journals.plos.org/plosone/s/figures

You may also use PLOS’s free figure tool, NAAS, to help you prepare publication quality figures: https://journals.plos.org/plosone/s/figures#loc-tools-for-figure-preparation.

NAAS will assess whether your figures meet our technical requirements by comparing each figure against our figure specifications.

# Author comment: Author response to Decision Letter 2

**DOI:** 10.1371/journal.pone.0356359.r004

7 Jul 2026

Responses to Reviewers’ comments

Dear editor and reviewers:

We extend our sincere gratitude for your valuable insights and professional guidance once again. On behalf of all co-authors, we appreciate your significant time and effort contributed to this manuscript. We have carefully reviewed all your feedback and made corresponding revisions. Each of your suggestions will be addressed in detail below. We hope the revised version now meets all publication requirements. Thank you once more for your support.

Title: FABP5 Regulates an Immunosuppressive Microenvironment in Hepatocellular Carcinoma Through the NF-κB/PD-L1 Axis.(Manuscript ID: PONE-D-26-15285R1 )

[Original title: FABP5 Shapes an Immunosuppressive Microenvironment in Hepatocellular Carcinoma Through the NF-κB/PD-L1 Axis.]

-Journal Requirements

1.Please provide the original files of western blot for checking.

Response: Thank you for the editor's reminder. All original, uncropped full membrane scans or film negatives of the western blot bands presented in the manuscript, along with molecular weight markers and sample labels, have been provided. These raw images have been compiled into a single PDF file labeled “original image of WB” and submitted as supplementary material in the submission system. Each membrane image is clearly annotated with the corresponding antibody information in accordance with journal policies. The files are fully accessible for verification purposes.We would like to once again express our sincere gratitude to the Academic Editor for the valuable and insightful comments.

2.Please review your reference list to ensure that it is complete and correct. If you have cited papers that have been retracted, please include the rationale for doing so in the manuscript text, or remove these references and replace them with relevant current references. Any changes to the reference list should be mentioned in the rebuttal letter that accompanies your revised manuscript. If you need to cite a retracted article, indicate the article’s retracted status in the References list and also include a citation and full reference for the retraction notice.

Response: Thank you for the valuable feedback provided by the editor. We have carefully reviewed our reference list to ensure that it is complete and correct. All cited articles have been verified, and we confirm that none of the references have been retracted. Therefore, no retraction notices are applicable, and no changes have been made to the reference list. The reference list remains as originally submitted and complies with the journal's requirements.

3.If you would like to make changes to your financial disclosure, please include your updated statement in your cover letter.

Response: Thank you for the editor's reminder. We confirm that our financial disclosure statement remains unchanged from the original submission, and no updates are necessary. Should the journal require any additional information, we are happy to provide it promptly.

4.If applicable, we recommend that you deposit your laboratory protocols in protocols.io to enhance the reproducibility of your results. Protocols.io assigns your protocol its own identifier (DOI) so that it can be cited independently in the future. For instructions see: https://journals.plos.org/plosone/s/submission-guidelines#loc-laboratory-protocols. Additionally, PLOS ONE offers an option for publishing peer-reviewed Lab Protocol articles, which describe protocols hosted on protocols.io. Read more information on sharing protocols at https://plos.org/protocols?utm_medium=editorial-email&utm_source=authorletters&utm_campaign=protocols.

Response: Our sincere gratitude to the editor for their constructive suggestions. We also acknowledge the journal’s recommendation to deposit laboratory protocols to protocols.io for improving research reproducibility. We will consider depositing our experimental protocols on this platform after formal acceptance of this work.

5.As the corresponding author, your ORCID iD is verified in the submission system and will appear in the published article. PLOS supports the use of ORCID, and we encourage all coauthors to register for an ORCID iD and use it as well. Please encourage your coauthors to verify their ORCID iD within the submission system before final acceptance, as unverified ORCID iDs will not appear in the published article. Only the individual author can complete the verification step; PLOS staff cannot verify ORCID iDs on behalf of authors.

Response: We sincerely thank the Academic Editor for the reminder regarding the ORCID iD verification requirement. In response, we confirm that the corresponding author’s ORCID iD has already been verified in the submission system. In addition, we have contacted all coauthors and strongly encouraged them to verify their own ORCID iDs through the submission system before final acceptance, in accordance with the journal’s policy.

-Reviewer 1

1. If the authors have adequately addressed your comments raised in a previous round of review and you feel that this manuscript is now acceptable for publication, you may indicate that here to bypass the “Comments to the Author” section, enter your conflict of interest statement in the “Confidential to Editor” section, and submit your "Accept" recommendation.

Reviewer 1: All comments have been addressed

Response: We are greatly thankful that the reviewer confirms all previous review concerns have been fully resolved. We have carefully polished the whole manuscript for minor editorial-level issues as the reviewer suggested, to further improve the readability and presentation quality of this work.

2. Is the manuscript technically sound, and do the data support the conclusions?

The manuscript must describe a technically sound piece of scientific research with data that supports the conclusions. Experiments must have been conducted rigorously, with appropriate controls, replication, and sample sizes. The conclusions must be drawn appropriately based on the data presented.

Reviewer 1: Yes

Response: We appreciate this positive recognition. All experiments in this study were designed with proper negative/positive controls, independent biological replicates and sufficient sample sizes. All experimental procedures were rigorously implemented following standard protocols, and all conclusions drawn are strictly supported by our original experimental data.

3. Has the statistical analysis been performed appropriately and rigorously?

Reviewer 1: Yes

Response: We thank the reviewer for the affirmation. All statistical methods applied in our study were selected rationally according to data distribution and experimental design. Statistical calculations, significance analysis and data visualization were completed with standardized statistical software, and we have double-checked all statistical results to ensure rigor and correctness.

4. Have the authors made all data underlying the findings in their manuscript fully available?

The PLOS Data policy requires authors to make all data underlying the findings described in their manuscript fully available without restriction, with rare exception (please refer to the Data Availability Statement in the manuscript PDF file). The data should be provided as part of the manuscript or its supporting information, or deposited to a public repository. For example, in addition to summary statistics, the data points behind means, medians and variance measures should be available. If there are restrictions on publicly sharing data—e.g. participant privacy or use of data from a third party—those must be specified.

Reviewer 1: Yes

Response: We appreciate this confirmation. All raw datasets, quantitative statistical source data and unprocessed experimental source images supporting our conclusions are preserved completely. In compliance with PLOS ONE data policy, we will provide original full-length western blot raw files for journal inspection in this revision submission, and we are ready to share all underlying original data upon reasonable request.

5. Is the manuscript presented in an intelligible fashion and written in standard English?

PLOS ONE does not copyedit accepted manuscripts, so the language in submitted articles must be clear, correct, and unambiguous. Any typographical or grammatical errors should be corrected at revision, so please note any specific errors here.

Reviewer 1: Yes

Response: We are grateful for this comment. During revision, we have carefully proofread the full text sentence by sentence to eliminate typographical mistakes, grammatical errors and awkward expressions, optimizing sentence logic and academic wording to conform to standard formal English writing requirements for biomedical research articles.

6. Review Comments to the Author

Please use the space provided to explain your answers to the questions above. You may also include additional comments for the author, including concerns about dual publication, research ethics, or publication ethics. (Please upload your review as an attachment if it exceeds 20,000 characters)

Reviewer 1: The revised manuscript has adequately addressed the major concerns raised during review and has been substantially improved. The remaining issues are minor and mainly editorial. I therefore support publication of the manuscript, subject to routine editorial polishing.

Response: We would like to express our sincere gratitude to the reviewer for the thorough and constructive evaluation of our manuscript over the two rounds of review. The insightful comments and suggestions have been extremely valuable in strengthening our work and improving the clarity of our presentation.

We are very pleased that the reviewer found our revisions satisfactory and confirmed that all concerns have been adequately addressed. We greatly appreciate the time and effort that the reviewer has dedicated to reviewing our submission. In accordance with your comment that only minor editorial-level issues remain, we have conducted comprehensive editorial polishing across the entire manuscript:

We carefully checked the consistency of terminology, abbreviations, gene/protein nomenclature (FABP5, NF-κB, PD-L1) throughout the full text to unify writing specifications;

We adjusted sentence structure, polished redundant expressions, and refined paragraph transitions to improve overall manuscript fluency and readability;

We re-examined figure legends, table annotations and reference formatting to match PLOS ONE formatting standards;

We cross-checked punctuation, spelling and grammatical details to eliminate trivial writing flaws.

After the above editorial revisions, we confirm there exists no dual publication risk, and all research was performed under complete compliance with relevant publication ethics and research ethics regulations.

Once again, we thank the reviewer for the positive feedback and invaluable guidance throughout this process.

7. PLOS authors have the option to publish the peer review history of their article (what does this mean?). If published, this will include your full peer review and any attached files.

If you choose “no”, your identity will remain anonymous but your review may still be made public.

Do you want your identity to be public for this peer review? For information about this choice, including consent withdrawal, please see our Privacy Policy.

Reviewer 1: No

Response: We respect the reviewer’s choice to remain anonymous in this peer review process, and we have no relevant inquiries regarding this setting.

Attachment Submitted filename: Response_to_reviewers_auresp_2.docx

# Editor report: Decision Letter 2

**DOI:** 10.1371/journal.pone.0356359.r005

**Authors:** Lanlan Chen

23 Jul 2026

PONE-D-26-15285R2 FABP5 Regulates an Immunosuppressive Microenvironment in Hepatocellular Carcinoma Through the NF-κB/PD-L1 Axis PLOS One

Dear Dr. An,

Thank you for submitting your manuscript to PLOS ONE. After careful consideration, we feel that it has merit but does not fully meet PLOS ONE’s publication criteria as it currently stands. Therefore, we invite you to submit a revised version of the manuscript that addresses the points raised during the review process.

Please submit your revised manuscript by Sep 06 2026 11:59PM. If you will need more time than this to complete your revisions, please reply to this message or contact the journal office at plosone@plos.org. When you're ready to submit your revision, log on to https://www.editorialmanager.com/pone/ and select the 'Submissions Needing Revision' folder to locate your manuscript file.

Please include the following items when submitting your revised manuscript:

- A letter that responds to each point raised by the academic editor and reviewer(s). You should upload this letter as a separate file labeled 'Response to Reviewers'.
- A marked-up copy of your manuscript that highlights changes made to the original version. You should upload this as a separate file labeled 'Revised Manuscript with Track Changes'.
- An unmarked version of your revised paper without tracked changes. You should upload this as a separate file labeled 'Manuscript'.

If you would like to make changes to your financial disclosure, please include your updated statement in your cover letter. Guidelines for resubmitting your figure files are available below the reviewer comments at the end of this letter.

If applicable, we recommend that you deposit your laboratory protocols in protocols.io to enhance the reproducibility of your results. Protocols.io assigns your protocol its own identifier (DOI) so that it can be cited independently in the future. For instructions see: https://journals.plos.org/plosone/s/submission-guidelines#loc-laboratory-protocols. Additionally, PLOS ONE offers an option for publishing peer-reviewed Lab Protocol articles, which describe protocols hosted on protocols.io. Read more information on sharing protocols at https://plos.org/protocols?utm_medium=editorial-email&utm_source=authorletters&utm_campaign=protocols.

As the corresponding author, your ORCID iD is verified in the submission system and will appear in the published article. PLOS supports the use of ORCID, and we encourage all coauthors to register for an ORCID iD and use it as well. Please encourage your coauthors to verify their ORCID iD within the submission system before final acceptance, as unverified ORCID iDs will not appear in the published article. *Only* the individual author can complete the verification step; PLOS staff *cannot* verify ORCID iDs on behalf of authors.

We look forward to receiving your revised manuscript.

Kind regards,

Lanlan Chen

Academic Editor

PLOS One

Journal Requirements:

If the reviewer comments include a recommendation to cite specific previously published works, please review and evaluate these publications to determine whether they are relevant and should be cited. There is no requirement to cite these works unless the editor has indicated otherwise.

Please review your reference list to ensure that it is complete and correct. If you have cited papers that have been retracted, please include the rationale for doing so in the manuscript text, or remove these references and replace them with relevant current references. Any changes to the reference list should be mentioned in the rebuttal letter that accompanies your revised manuscript. If you need to cite a retracted article, indicate the article’s retracted status in the References list and also include a citation and full reference for the retraction notice.

Additional Editorial Staff Requirements:

Please provide the original, uncropped and minimally adjusted images supporting all blot and gel results reported in an article’s figures and supporting information files (see for more information: https://journals.plos.org/plosone/s/figures#loc-blot-and-gel-reporting-requirements).

[Note: HTML markup is below. Please do not edit.]

Reviewers' comments:

[NOTE: If reviewer comments were submitted as an attachment file, they will be attached to this email and accessible via the submission site. Please log into your account, locate the manuscript record, and check for the action link "View Attachments". If this link does not appear, there are no attachment files.]

To ensure your figures meet our technical requirements, please review our figure guidelines: https://journals.plos.org/plosone/s/figures

You may also use PLOS’s free figure tool, NAAS, to help you prepare publication quality figures: https://journals.plos.org/plosone/s/figures#loc-tools-for-figure-preparation.

NAAS will assess whether your figures meet our technical requirements by comparing each figure against our figure specifications.

# Author comment: Author response to Decision Letter 3

**DOI:** 10.1371/journal.pone.0356359.r006

26 Jul 2026

Responses to Reviewers’ comments

Dear editor and reviewers:

We extend our sincere gratitude for your valuable insights and professional guidance once again. On behalf of all co-authors, we appreciate your significant time and effort contributed to this manuscript. We have carefully reviewed all your feedback and made corresponding revisions. Each of your suggestions will be addressed in detail below. We hope the revised version now meets all publication requirements. Thank you once more for your support.

Title: FABP5 Regulates an Immunosuppressive Microenvironment in Hepatocellular Carcinoma Through the NF-κB/PD-L1 Axis.(Manuscript ID: PONE-D-26-15285R2 )

[Original title: FABP5 Shapes an Immunosuppressive Microenvironment in Hepatocellular Carcinoma Through the NF-κB/PD-L1 Axis.]

-Journal Requirements

1.If you would like to make changes to your financial disclosure, please include your updated statement in your cover letter. Guidelines for resubmitting your figure files are available below the reviewer comments at the end of this letter.

Response: We are grateful for the opportunity to revise our work. We have reviewed our financial disclosure statement and confirm that no changes are needed. The original statement remains accurate. We hope that the revised manuscript now meets the journal's requirements. Please do not hesitate to let us know if any further revisions are needed.

2.If the reviewer comments include a recommendation to cite specific previously published works, please review and evaluate these publications to determine whether they are relevant and should be cited. There is no requirement to cite these works unless the editor has indicated otherwise.

Response: Thank you for the valuable feedback provided by the editor. The reviewers did not provide any specific recommendations for additional citations. We have therefore maintained our reference list as originally submitted, with updates made only where necessary to address the scientific content of the reviews.

3.Please review your reference list to ensure that it is complete and correct. If you have cited papers that have been retracted, please include the rationale for doing so in the manuscript text, or remove these references and replace them with relevant current references. Any changes to the reference list should be mentioned in the rebuttal letter that accompanies your revised manuscript. If you need to cite a retracted article, indicate the article’s retracted status in the References list and also include a citation and full reference for the retraction notice.

Response: Thank you for the editor’s reminder. We have carefully reviewed our entire reference list. We confirm that none of the cited papers have been retracted. All references are complete, accurate, and relevant to the content of our manuscript.

4.Please provide the original, uncropped and minimally adjusted images supporting all blot and gel results reported in an article’s figures and supporting information files (see for more information: https://journals.plos.org/plosone/s/figures#loc-blot-and-gel-reporting-requirements).

Response: Our sincere gratitude to the editor for their constructive suggestions. We have compiled all original, uncropped, and minimally adjusted images supporting the blot and gel results presented in our manuscript’s figures and supporting information. These have been assembled into a single PDF file named S1 raw_images. This file has been uploaded as a Supporting Information file as per the journal’s guidelines. We confirm that all images comply with PLOS ONE’s blot and gel reporting requirements.

-Reviewer

Response to reviewers: We would like to express our sincere gratitude to the editor and the reviewers for taking the time to evaluate our manuscript. We greatly appreciate the thoughtful assessment of our work.The reviewers did not raise any specific concerns or requests for revision regarding the scientific content of our manuscript. We are very encouraged by this positive feedback.We trust that the revised version now meets the journal's standards for publication. Should the reviewers or editors have any further suggestions, we would be happy to address them promptly.

Attachment Submitted filename: Response_to_reviewers_auresp_3.docx

# Editor report: Decision Letter 3

**DOI:** 10.1371/journal.pone.0356359.r007

**Authors:** Lanlan Chen

3 Aug 2026

FABP5 Regulates an Immunosuppressive Microenvironment in Hepatocellular Carcinoma Through the NF-κB/PD-L1 Axis

PONE-D-26-15285R3

Dear Dr. An,

We’re pleased to inform you that your manuscript has been judged scientifically suitable for publication and will be formally accepted for publication once it meets all outstanding technical requirements.

Within one week, you’ll receive an e-mail detailing the required amendments. When these have been addressed, you’ll receive a formal acceptance letter and your manuscript will be scheduled for publication.

An invoice will be generated when your article is formally accepted. Please note, if your institution has a publishing partnership with PLOS and your article meets the relevant criteria, all or part of your publication costs will be covered. Please make sure your user information is up-to-date by logging into Editorial Manager at Editorial Manager® and clicking the ‘Update My Information' link at the top of the page. For questions related to billing, please contact billing support.

If your institution or institutions have a press office, please notify them about your upcoming paper to help maximize its impact. If they’ll be preparing press materials, please inform our press team as soon as possible -- no later than 48 hours after receiving the formal acceptance. Your manuscript will remain under strict press embargo until 2 pm Eastern Time on the date of publication. For more information, please contact onepress@plos.org.

Kind regards,

Lanlan Chen

Academic Editor

PLOS One

Additional Editor Comments (optional):

Thanks a lot for addressing all concerns.

Reviewers' comments:

# Editor report: Acceptance letter

**DOI:** 10.1371/journal.pone.0356359.r008

**Authors:** Lanlan Chen

PONE-D-26-15285R3

PLOS One

Dear Dr. An,

I'm pleased to inform you that your manuscript has been deemed suitable for publication in PLOS One. Congratulations! Your manuscript is now being handed over to our production team.

At this stage, our production department will prepare your paper for publication. This includes ensuring the following:

* All references, tables, and figures are properly cited

* All relevant supporting information is included in the manuscript submission,

* There are no issues that prevent the paper from being properly typeset

You will receive further instructions from the production team, including instructions on how to review your proof when it is ready. Please keep in mind that we are working through a large volume of accepted articles, so please give us a few days to review your paper and let you know the next and final steps.

Lastly, if your institution or institutions have a press office, please let them know about your upcoming paper now to help maximize its impact. If they'll be preparing press materials, please inform our press team within the next 48 hours. Your manuscript will remain under strict press embargo until 2 pm Eastern Time on the date of publication. For more information, please contact onepress@plos.org.

You will receive an invoice from PLOS for your publication fee after your manuscript has reached the completed accept phase. If you receive an email requesting payment before acceptance or for any other service, this may be a phishing scheme. Learn how to identify phishing emails and protect your accounts at https://explore.plos.org/phishing.

If we can help with anything else, please email us at customercare@plos.org.

Thank you for submitting your work to PLOS ONE and supporting open access.

Kind regards,

PLOS ONE Editorial Office Staff

on behalf of

M.D Lanlan Chen

Academic Editor

PLOS One
